# Kidney Dysfunction Is Associated with Thrombosis and Disease Severity in Myeloproliferative Neoplasms: Implications from the German Study Group for MPN Bioregistry

**DOI:** 10.3390/cancers13164086

**Published:** 2021-08-13

**Authors:** Judith Gecht, Ioannis Tsoukakis, Kim Kricheldorf, Frank Stegelmann, Martine Klausmann, Martin Griesshammer, Holger Schulz, Wiebke Hollburg, Joachim R. Göthert, Katja Sockel, Florian H. Heidel, Norbert Gattermann, Christoph Maintz, Haifa K. Al-Ali, Uwe Platzbecker, Richard Hansen, Mathias Hänel, Stefani Parmentier, Martin Bommer, Heike L. Pahl, Fabian Lang, Martin Kirschner, Susanne Isfort, Tim H. Brümmendorf, Konstanze Döhner, Steffen Koschmieder

**Affiliations:** 1 Department of Hematology, Oncology, Hemostaseology and SCT, Faculty of Medicine, RWTH Aachen University, 52074 Aachen, Germany; jgecht@ukaachen.de (J.G.); Ioannis.Tsoukakis@kgu.de (I.T.); kkricheldorf@ukaachen.de (K.K.); mkirschner@ukaachen.de (M.K.); sisfort@ukaachen.de (S.I.); tbruemmendorf@ukaachen.de (T.H.B.); 2 Center for Integrated Oncology Aachen Bonn Cologne Düsseldorf (CIO ABCD), 52074 Aachen, Germany; gattermann@med.uni-duesseldorf.de; 3 Department of Medicine, Hematology/Oncology, Goethe-University, 60590 Frankfurt am Main, Germany; Fabian.Lang@kgu.de; 4 Department of Internal Medicine III, University Hospital of Ulm, 89081 Ulm, Germany; frank.stegelmann@uniklinik-ulm.de (F.S.); konstanze.doehner@uniklinik-ulm.de (K.D.); 5 Studienzentrum Aschaffenburg, 63739 Aschaffenburg, Germany; mk@klausmann.de; 6 Johannes Wesling Medical Center, University Clinic for Hematology, Oncology, Hemostaseology, and Palliative Care (UKRUB), University of Bochum, 32429 Minden, Germany; Martin.Griesshammer@muehlenkreiskliniken.de; 7 Oncological Practice, 50226 Frechen, Germany; hschulz@pioh.de; 8 HOPA-Hämatologisch-Onkologische Praxis Altona, 22767 Hamburg, Germany; wiebke.hollburg@hopa-hamburg.de; 9 Department of Hematology and Stem Cell Transplantation, University Hospital Essen, 45147 Essen, Germany; Joachim.Goethert@uk-essen.de; 10 Medical Clinic and Policlinic I, University Hospital Carl Gustav Carus, TU Dresden, 01307 Dresden, Germany; katja.sockel@uniklinikum-dresden.de; 11 Innere Medizin C, Universitätsmedizin Greifswald, 17475 Greifswald, Germany; florian.heidel@uni-greifswald.de; 12 Department of Hematology/Oncology, Clinic of Internal Medicine II, Jena University Hospital, 07747 Jena, Germany; 13 Department of Hematology, Oncology and Clinical Immunology, Heinrich Heine University Düsseldorf, 40225 Düsseldorf, Germany; 14 MWZ-West Hematology-Oncology, 52146 Würselen, Germany; c.maintz@aachen-onkologie.de; 15 Department of Hematology/Oncology, University Hospital Halle, 06120 Halle, Germany; haifa.al-ali@uk-halle.de; 16 Department of Hematology and Cellular Therapy, Medical Clinic and Policlinic I, Leipzig University Hospital, 04103 Leipzig, Germany; Uwe.Platzbecker@medizin.uni-leipzig.de; 17 Oncological Practice Dres. Hansen & Reeb, 67655 Kaiserslautern, Germany; richard.hansen@pfalz-onko.de; 18 Department of Internal Medicine III, Klinikum Chemnitz, 09116 Chemnitz, Germany; m.haenel@skc.de; 19 Department of Hematology and Oncology, Rems-Murr-Klinikum Winnenden, 71364 Winnenden, Germany; Stefani.Parmentier@claraspital.ch; 20 Onkologie/Hämatologie, Claraspital Tumorzentrum Basel, 4058 Basel, Switzerland; 21 Department of Hematology, Oncology, Infectious Diseases and Palliative Care, Alb-Fils-Kliniken, 73035 Göppingen, Germany; Martin.Bommer@af-k.de; 22 Department of Medicine I, Hematology and Oncology, University Medical Center Freiburg, Faculty of Medicine, University of Freiburg, 79106 Freiburg, Germany; heike.pahl@uniklinik-freiburg.de

**Keywords:** myeloproliferative neoplasms (MPN), renal dysfunction, chronic kidney disease, thrombosis, thromboembolism, bleeding, JAK2V617F, essential thrombocythemia (ET), polycythemia vera (PV), primary myelofibrosis (PMF)

## Abstract

**Simple Summary:**

In patients with myeloproliferative neoplasms (MPN) and in patients with kidney dysfunction, a higher rate of thrombosis has been reported compared with the general population. Furthermore, MPN patients are more prone to develop kidney dysfunction. In our study, we assessed the importance of specific risk factors for kidney dysfunction and thrombosis in MPN patients. We found that the rate of thrombosis is correlated with the degree of kidney dysfunction, especially in myelofibrosis. Significant associations for kidney dysfunction included arterial hypertension, MPN treatment, and increased inflammation, and those for thrombosis comprised arterial hypertension, non-excessive platelet counts, and antithrombotic therapy. The identified risk factor associations varied between MPN subtypes. Our data suggest that kidney dysfunction in MPN patients is associated with an increased risk of thrombosis, mandating closer monitoring, and, possibly, early thromboprophylaxis.

**Abstract:**

Inflammation-induced thrombosis represents a severe complication in patients with myeloproliferative neoplasms (MPN) and in those with kidney dysfunction. Overlapping disease-specific attributes suggest common mechanisms involved in MPN pathogenesis, kidney dysfunction, and thrombosis. Data from 1420 patients with essential thrombocythemia (ET, 33.7%), polycythemia vera (PV, 38.5%), and myelofibrosis (MF, 27.9%) were extracted from the bioregistry of the German Study Group for MPN. The total cohort was subdivided according to the calculated estimated glomerular filtration rate (eGFR, (mL/min/1.73 m^2^)) into eGFR1 (≥90, 21%), eGFR2 (60–89, 56%), and eGFR3 (<60, 22%). A total of 29% of the patients had a history of thrombosis. A higher rate of thrombosis and longer MPN duration was observed in eGFR3 than in eGFR2 and eGFR1. Kidney dysfunction occurred earlier in ET than in PV or MF. Multiple logistic regression analysis identified arterial hypertension, MPN treatment, increased uric acid, and lactate dehydrogenase levels as risk factors for kidney dysfunction in MPN patients. Risk factors for thrombosis included arterial hypertension, non-excessive platelet counts, and antithrombotic therapy. The risk factors for kidney dysfunction and thrombosis varied between MPN subtypes. Physicians should be aware of the increased risk for kidney disease in MPN patients, which warrants closer monitoring and, possibly, early thromboprophylaxis.

## 1. Introduction

Patients with myeloproliferative neoplasms (MPNs) are at increased risk of developing myelofibrosis as well as vascular complications such as thrombosis or severe hemorrhage [1,2,3,4,5,6], all of which are associated with inferior survival. Risk factors for thrombosis in MPN patients include advanced age (e.g., ≥60 years), history of thrombosis, leukocytosis, and JAK2V617F positivity [5,7,8,9,10,11,12]. In the general population, there is evidence for a link between thrombosis, inflammation, chronic kidney disease (CKD), and survival [13,14,15,16,17,18,19,20]. CKD occurs significantly more frequently in MPN patients than in the general population, as documented by renal involvement in several case studies [21,22,23] as well as larger monocenter [24,25] or oligocentric studies [26,27]. These studies have shown that CKD is associated with thrombosis in MPN patients [26] and that kidney function declines with MPN duration beyond the expected age-related decline, suggesting that the MPN itself has a deteriorating impact on kidney function [2,24,26]. These studies also suggested that inflammatory factors are involved in kidney dysfunction in these patients [24,25], providing a potential functional link between MPN and CKD pathogenesis. In keeping with this hypothesis, patients with primary myelofibrosis (PMF) may experience improvement of kidney function when treated with the JAK inhibitor ruxolitinib (RUX) [28]. However, whether cytoreductive therapy for MPN improves renal dysfunction overall remains a matter of current debate: while one study showed an increase in the estimated glomerular filtration rate (eGFR) during hydroxyurea treatment (HU) in polycythemia vera (PV) patients [25], this was not seen in another study [29], where HU appeared to be beneficial in PMF, but it was not the case in PV or in essential thrombocythemia (ET) patients. A third study [24] found that HU treatment did not significantly affect progression of kidney dysfunction during the course of MPN disease. In summary, the above-mentioned studies suggest an association between CKD and thrombosis in MPN patients over time. However, these analyses were limited by the small number of centers, by the limited size of the cohorts, and by conflicting results on the severity of kidney dysfunction in each MPN subgroup. Furthermore, the classification of kidney dysfunction was restricted to eGFR calculation, which includes age, thereby possibly obscuring a possible relationship between thrombosis, CKD, and MPN. The present study aims to investigate the relationship between CKD and thrombosis separately in ET, PV, and myelofibrosis (MF) patients in a large multicenter cohort and to examine the association of MPN duration and kidney dysfunction, as measured by eGFR or isolated creatinine serum levels. Moreover, our study aimed at analyzing the risk factors for CKD and thrombosis, the impact of different treatment modalities, and the effect of CKD on patients’ survival.

## 2. Materials and Methods

### 2.1. Study Design and Study Population

The present retrospective multicenter study is a secondary analysis of existing data collected in the ongoing prospective MPN bioregistry study of the German Study Group for MPN (GSG-MPN bioregistry), which has recruited patients since 2012. A total of 1979 MPN patients from 52 centers, included between 2012 and December 2019, were screened. Diagnosis of ET, PV, or MF (comprising PMF, post-ET-PMF, and post-PV-MF) was required, as defined by the World Health Organization (WHO) classifications of 2008 [30], 2001 (for patients diagnosed before 2008), or the International Working Group-Myeloproliferative Neoplasms Research and Treatment (IWG-MRT) criteria (for post-ET-MF and post-PV-MF). Patients with other MPNs or missing serum creatinine measurements (*n* = 559) were excluded, resulting in a study sample size of 1420 patients.

The following risk factors, hypothesized to be associated with thrombosis, bleeding, and CKD in MPN patients, were collected: presence/type of thrombosis, severe hemorrhage, comorbidities (diabetes mellitus, arterial hypertension, and hyperlipidemia), blood cell parameters (leukocytes (G/L), platelets (G/L), monocytes (G/L), neutrophils (G/L)), JAK2V617F and CALR status, and parameters of proliferative activity and inflammation (lactate dehydrogenase (LDH, U/L), C-reactive protein (CRP, mg/L), uric acid (mg/dL)). For each risk factor, median values were used in order to classify patients into a high or a low group. Additionally, MPN duration, MPN-specific therapy (HU, RUX, and “other MPN treatment”, i.e., anagrelide, interferon, immunomodulatory drugs (IMIDs)), and antithrombotic therapy were documented. Finally, the time from diagnosis to last follow-up (FU) or date of death was assessed for survival analysis in relation to kidney dysfunction, and the Eastern Cooperative Oncology Group Performance Status (ECOG PS) was collected as a quantification of patients’ capability to engage in activities of daily living (ADL).

### 2.2. Assessment of Kidney Function

Kidney function was calculated using the simplified Modification of Diet in Renal Disease (MDRD) formula to obtain the eGFR (mL/min/1.73 m^2^) [31]:

eGFR = 175 × Scr ^−1.154^ × age ^−0.203^ × 0.742 [if female] × 1.212 [if black]

where Scr is serum creatinine (mg/dL).

According to the Kidney Disease Improving Global Outcomes (KDIGO) 2012 guidelines [32], CKD is defined as abnormalities of kidney structure or function, if they have been present for over 3 months. In patients with a GFR below 60 mL/min/1.73m^2^, which persists over 3 months, CKD is confirmed, regardless of the presence of albuminuria [32]. In the present study, patients were subdivided into three eGFR subgroups, termed eGFR1, eGFR2, and eGFR3, according to their calculated eGFR: eGFR1: ≥90 mL/min/1.73 m^2^; eGFR2: 60–89 mL/min/1.73 m^2^; and eGFR3: <60 mL/min/1.73 m^2^, respectively. As an indicator of age-independent kidney function, the serum creatinine level was used.

### 2.3. Statistical Methods

Descriptive data are presented as percentage or median including quartiles (Q1, Q3) and were analyzed using χ^2^-test (Pearson’s χ^2^ or Fisher’s exact test for 2by2 contingency tables and larger tables with expected cell counts <5, respectively) or Wilcoxon–Mann–Whitney/Kruskal–Wallis test, as appropriate. Univariate and multiple logistic regression analyses were applied for associations, expressed as odds ratios (ORs) with 95% confidence intervals (95% CIs), between the occurrence of thrombosis, severe bleeding, and decreased kidney function in relation to the above-mentioned risk factors. Age and sex were not included in the regression analyses of kidney function, since they are components of the eGFR formula. Risk factors with significance shown in univariate analyses were included in the basic models in multiple regression analyses. In order to evaluate and quantify progression of kidney function in relation to MPN duration, logistic regression models were used. The impact of kidney function on overall survival was estimated according to Kaplan–Meier and assessed by log-rank testing and Cox regression models. Data analysis was performed using SAS software 9.4. [33]. Statistical significance was indicated with a two-tailed *p* < 0.05. Multiple comparisons were adjusted by Bonferroni correction.

## 3. Results

### 3.1. General Characteristics of Study Cohort

Among the 1420 patients, 39% of the patients had ET, 34% had PV, and 28% had MF; 49% of the patients were male, with the expected lower percentage in ET and higher percentage in MF (Table 1). JAK2V617F status was documented in 1315 patients of whom 73% were positive; of the 509 patients analyzed for CALR mutation, 27% were positive (Table 1 and Table S1). At the time of kidney function assessment, 40% of the patients were treated with HU, 19% with RUX, and 21% of the patients were treated with anagrelide, IMIDs, or interferon, respectively, with expected differences among the MPN subtypes, reflecting the drug approval; 19%, 12%, and 4% had a history of arterial thrombosis, venous thrombosis, or severe hemorrhage, respectively. Diabetes mellitus was diagnosed in 8% of the patients, 49% had arterial hypertension, and 12% hyperlipidemia (Table 1). In the total cohort, ECOG PS 0 was assigned to 69% of all patients, while 27% had ECOG PS 1. The proportion of patients with ECOG PS 1 was significantly higher in MF than in ET, being 34% and 22%, respectively (Table 1). The patients’ median age at diagnosis was 57 years, with ET patients being significantly younger than PV and MF patients. Median MPN duration at time of creatinine testing was 3.5 years and was shortest in MF patients (Table 1). Relevant laboratory parameters are depicted in Table 1.

**Figure 1:**
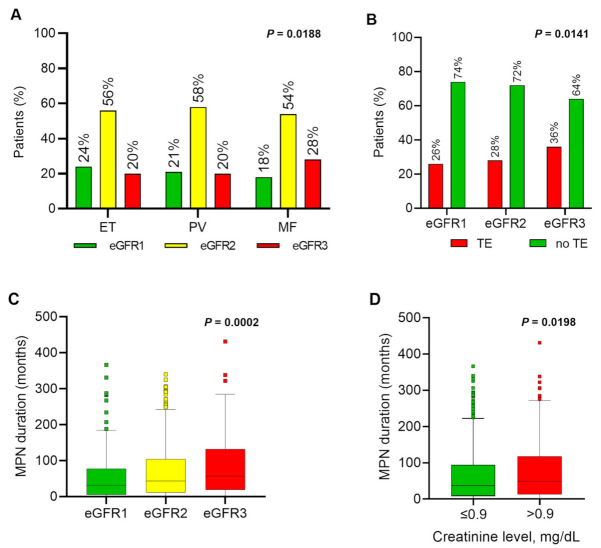
Distribution of kidney dysfunction, thrombosis, and MPN duration across all patients (pts; *n* = 1420). (**A**) Distribution (%) of kidney function groups eGFR1 (≥90 mL/min/1.73 m^2^), eGFR2 (60–89 mL/min/1.73 m^2^), or eGFR3 (<60 mL/min/1.73 m^2^) across the MPN subtypes ET, PV, and MF; (**B**) Percentage of pts with (yes) or without (no) a history of thromboembolic events (“thrombosis”) among the eGFR1,2,3 groups; (**C**) MPN duration in months from diagnosis to renal function assessment in the eGFR1,2,3 groups. In addition, median MPN duration is indicated for each eGFR group; (**D**) MPN duration in months from diagnosis to renal function assessment in the group of patients with a creatinine level at or below the median (≤0.9 mg/dL) or above the median (>0.9 mg/dL). In addition, median MPN duration is indicated for both groups. * Wilcoxon–Mann–Whitney test.

Of all the patients, 21%, 56%, and 22% exhibited an eGFR of ≥90 (eGFR1), 60–89 (eGFR2), and <60 (eGFR3) mL/min/1.73 m^2^, respectively, at baseline (time of inclusion into the registry or routine follow-up) (Table 1). Median eGFR in ET, PV, and MF was 77, 74, and 72 mL/min/1.73 m^2^, respectively. In MF, a higher fraction of patients fell into the eGFR3 category compared with ET and PV (Table 1; Figure 1A). A total of 55% of patients were older than 60 years at creatinine measurement, and the highest proportion of these patients fell into eGFR3 (Table S1). In the total cohort, 29% of the patients had a thrombotic event and 4% had severe bleeding. The rate of thrombosis was higher in PV (34%) than in ET (26%) or MF (28%).

### 3.2. Differences among the eGFR Subgroups 

#### 3.2.1. Thrombosis and Bleeding

In Tables S1–S4, the specific risk factors for CKD in each eGFR group are presented for the overall cohort (Table S1) and separately for ET, PV, and MF (Tables S2–S4). In the total cohort, the rate of thrombosis was significantly higher in the eGFR3 group (36%) vs. eGFR1 (26%) or eGFR2 groups (28%) (Table S1). Significantly more thromboses occurred in eGFR3 in PV (45%) as compared to ET (31%) or MF (32%) (Tables S2–S4; Figure 1B). No differences in bleeding events were found across the eGFR groups (Table S1).

#### 3.2.2. Comorbidities

In the total cohort, diabetes mellitus, arterial hypertension, and hyperlipidemia were significantly more prevalent in eGFR3 than in eGFR2 or eGFR1 groups (Tables S1–S4). However, this was true for ET (Table S2) and MF (Table S4) but not for PV patients (Table S3), although the overall percentages of diabetics in ET, PV, and MF were similar (6.4% to 9.5%) (Tables S2–S4). In line with these data, more patients in the eGFR3 had a poorer ECOG PS than in the eGFR2 group, particularly in MF (Tables S1–S4).

#### 3.2.3. Uric Acid and LDH Levels

As expected, the proportion of patients with elevated uric acid levels was higher in eGFR3 than in eGFR1 (Tables S2–S4), in keeping with elevated uric acid levels due to decreased kidney function. However, uric acid (and LDH) levels in eGFR1 were significantly higher in PV and MF than in ET, indicative of higher cell turnover. This was consistent with the positive correlation between uric acid levels and leukocytosis and LDH, which was also seen in the absence of severe kidney dysfunction (eGFR1/eGFR2 groups) (Table S5).

#### 3.2.4. Blood Counts and C-Reactive Protein

Overall, elevated leukocyte, neutrophil, and monocyte counts were significantly associated with decreased kidney function, while elevated platelet counts were less frequent in these patients (Table S1). Differences between the MPN subtypes were identified, with MF showing the strongest association between leukocytosis/neutrophilia and decreased kidney function, while there were no significant associations between blood counts and kidney function in PV (Tables S2–S4). Elevated CRP levels were significantly associated with kidney dysfunction in the entire cohort.

#### 3.2.5. Driver Mutations

The presence of the JAK2V617F mutation has been associated with an increased risk of thrombosis in ET [34,35], and the mechanism involved enhanced activation of platelets, leukocytes, and endothelial cells as well as prothrombotic soluble factors [36,37,38]. In our overall cohort, JAK2V617F was not associated with kidney dysfunction; however, CALR mutations were significantly less frequent in eGFR3 (Table S1). When analyzing the effects separately for each MPN subtype, significant associations were found between JAK2V617F positivity and kidney dysfunction in ET but neither in PV nor in MF (Tables S2–S4). Interestingly, leukocyte counts were also higher in JAKV617F positive ET patients than CALR-mutant ET patients [35].

#### 3.2.6. MPN Duration

A longer MPN duration was found in eGFR3 than in eGFR2 and eGFR1. When patients were categorized into two groups above and below the median duration of 42 months, more patients with a longer duration fell into eGFR3 and eGFR2 compared with eGFR1 (Tables S6 and S7; Figure 1C). Adjusting for the influence of patients’ age in the calculation of eGFR by focusing only on creatinine levels, MPN duration was significantly longer in patients with an elevated creatinine level (cut-off: 0.9 mg/dL; Table S8; Figure 1D). Likewise, patients with an MPN duration longer than 42 months were more prevalent in the high than in the low creatinine group (Table S9). Univariate logistic regression revealed associations for a longer MPN duration with ET and PV vs. MF, absence of diabetes mellitus, leukocyte counts of 8.4 G/L or lower, arterial hypertension, MPN therapy (besides RUX), and acetylsalicylic acid (ASA) (Table S10). Upon multivariable regression, risk factors differed between MPN subtypes. In ET, arterial hypertension, treatment with HU, and other MPN treatment remained significant. In PV, absence of diabetes mellitus and RUX treatment were identified as additional relevant risk factors. In MF, only HU-treatment or other MPN treatments were associated with a longer MPN duration (Table S10). 

### 3.3. Risk Factors for Decreased Kidney Function

Each of the expected risk factors for decreased kidney function was identified as a significant correlate in univariate logistic regression. This was the case for kidney dysfunction in general, but also remained true across the specific eGFR groups (Table 2 displays ORs, 95%CIs, and *P*). MF, co-existing diabetes mellitus, hyperlipidemia, leukocytes > 8.4 G/L, neutrophils > 5.48 G/L, monocytes > 0.55 G/L, LDH > 267.5 U/L, CRP levels > 1.4 mg/L, and treatment with RUX and anagrelide/IMIDs/interferon showed significant ORs when contrasting eGFR3 to eGFR2 or eGFR1. As stated above, MPN duration > 42 months was associated with kidney dysfunction. Arterial hypertension, uric acid levels > 5.7 mg/dL, and HU treatment were significant risk factors in all eGFR groups. Associations of age and sex with kidney function were not examined, since these factors are part of the eGFR formula. In multiple logistic regression analysis, HU treatment was significantly associated with kidney dysfunction (Table 2). When comparing eGFR3 vs. eGFR1, arterial hypertension, uric acid levels > 5.7 mg/dL, and each of the three MPN treatments were significant correlates of kidney dysfunction. The eGFR groups, eGFR3 and eGFR2, differed regarding the relevance of arterial hypertension, elevated levels of uric acid and LDH, and MPN treatment (besides RUX).

### 3.4. Risk Factors for Developing Thrombosis and Bleeding in the Presence of Kidney Dysfunction

Table S11 shows the frequencies of thrombosis stratified for the presence and absence of each risk factor across each eGFR group, and Table S12 displays the distribution of arterial and venous thromboses in each group. The higher rate of thrombosis in the eGFR3 group was significant for female but not for male patients and for younger (≤60 years) but not for older patients, although a similar trend was observed for both groups. The higher thrombosis rate in the eGFR3 group was independent of the JAK2V617F mutation (Table S11). Elevated counts of leukocytes, platelets, neutrophils, and monocytes were associated with a higher frequency of thrombosis within the eGFR3 group. Univariate analysis (Table 3) demonstrated JAK2V617F positivity, platelets ≤492 G/L, eGFR3, arterial hypertension, and hyperlipidemia as significant risk factors for thrombosis. More thromboses were also found for patients with anti-MPN treatment and antithrombotic therapy. In multiple logistic regression analysis, arterial hypertension, platelets, and antithrombotic therapy remained significant in the total cohort. Risk factor analysis for each MPN subtype is included in Table 3. Univariate risk factors for severe bleeding were male sex, platelet counts ≤492 G/L, and elevated LDH, with only LDH remaining upon multivariable analysis (Table 4).

### 3.5. Treatment with Hydroxyurea, Ruxolitinib, and Anticoagulants Are Associated with Kidney Dysfunction

Patients´ median age when starting MPN treatment was 52, 60, and 67 years in eGFR1, eGFR2, and eGFR3, respectively, with significant differences between all eGFR groups. Compared to patients with no therapy (watch-and-wait (WW)), treatment with HU only, RUX only, and multiple lines of MPN therapies were associated with kidney dysfunction (Table 5). Significantly lower median eGFR values were found for HU in ET, for HU and RUX in MF, and for multiple lines of MPN therapy in the overall cohort (Table 5). Patients having switched from HU to RUX or vice versa were not included in the HUonly or RUX-only cohorts. In the total cohort, the frequency of patients in eGFR3 was higher with HU or RUX treatment than no treatment (Table S13). These effects remained significant for HU in the ET and MF subtypes, and for RUX in the MF subtype, while, again, no significant association between treatment and kidney dysfunction was found for PV (Table S13). Median eGFR was comparable in patients regardless of whether they received anticoagulant therapy, antiplatelet agents, or watchful management (Table 6), but anticoagulant use was more frequent in eGFR3 than in eGFR1/eGFR2 patients (Table S14). Together, these results suggest that, most likely, HU, RUX, and anticoagulant therapy was started in patients with a higher risk for kidney dysfunction, including those with advanced age.

### 3.6. Survival

**Figure 2:**
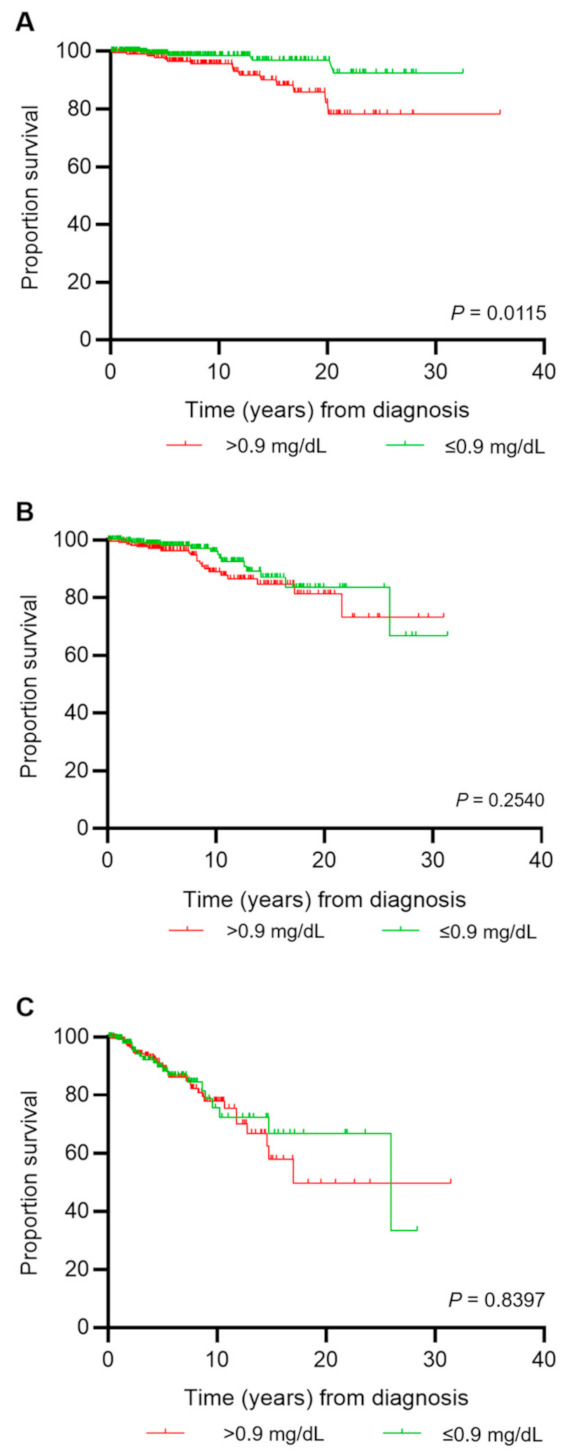
Overall survival according to creatinine level ≤0.9 mg/dL (green) or >0.9 mg/dL (red) at entry into the registry. Creatinine rather than eGFR was used for survival analysis, since the eGFR formula contains age as one of the variables. (**A**) Survival analysis for pts with essential thrombocythemia (ET); (**B**) survival analysis for pts with polycythemia vera (PV); (**C**) survival analysis for pts with myelofibrosis (MF), including pts with primary MF, post-PV MF, and post-ET MF.

In order to evaluate the association of kidney dysfunction with patients’ survival, we analyzed overall survival after MPN diagnosis in the two creatinine groups (>0.9 mg/dL and ≤0.9 mg/dL) (Figure 2A–C). Overall survival differed significantly between the creatine groups and when stratified for MPN subtype; this was confirmed in ET but not in PV or MF. Accordingly, Cox regression identified a higher risk of death in the creatinine group >0.9 mg/dL compared with ≤0.9 mg/dL in ET (hazard ratio (HR) = 3.4, 95% CI (1.2–9.4), *p* = 0.0174) but not in PV or MF.

## 4. Discussion

In this retrospective multicenter analysis of the German GSG-MPN bioregistry, the majority of patients had an eGFR between 60 and 89 mL/min/1.73 m^2^. Compared with the general population [39,40,41], median eGFR was lower in our patient cohort but similar to a previous study in MPN patients [24], while another study had reported higher levels [25] (74, 73, and 82 mL/min/1.73 m^2^, respectively). The fraction of patients with kidney dysfunction (eGFR < 60 mL/min/1.73 m^2^) was 22.3% in our cohort and 29% [24], 15.4% [25], and 27% [26] in the previous studies, respectively. Mean age at analysis in our cohort (62 years) was similar to the previous cohorts (63.2 [24], 62 [25], and 64 years [26], respectively) and thus did not account for the eGFR differences between the cohorts.

Our data revealed a higher prevalence of kidney dysfunction in MF patients compared with ET or PV, indicating that MF has a higher impact on kidney function and that this was not simply due to a higher age or diabetes prevalence (see Table 1). This is a novel finding, since previous studies either reported similar frequencies of CKD among the three subtypes ET/PV/MF [24,25] or did not include MF patients [26]. The more severe kidney dysfunction in MF patients may also account for the higher rate of ECOG PS 1 vs. 0 in this patient group, reflecting impairments in ADL.

When comparing more severe (eGFR < 60 mL/min/1.73 m^2^) to milder (eGFR ≥ 60 mL/min/1.73 m^2^) kidney dysfunction, the most relevant factors accompanying decreased function were co-existing diabetes mellitus, arterial hypertension, hyperlipidemia, elevated levels of uric acid, leukocytes, neutrophils, monocytes, LDH, CRP, and MPN therapy (Table 2). A longer MPN duration was associated with a higher prevalence of kidney dysfunction in our cohort, when using either eGFR or creatinine levels (Figure 1C,D). Thus, established risk factors for developing CKD in the general population [42,43] were also relevant in MPN patients overall, but we found that diabetes, arterial hypertension, and hyperlipidemia may be less important in PV than in ET and MF (Tables S2–S4). Moreover, inflammatory biomarkers were associated with decreased kidney function in our cohort, in keeping with findings in previous MPN studies [25,26]. Chronic inflammation is relevant for MPN pathogenesis [25,44], and CKD itself is also associated with (chronic) inflammation [45,46]. Our study supports the concept that MPN-induced inflammation adversely affects kidney function and shows that inflammatory cells are more important factors for kidney dysfunction than CRP levels.

In PV, co-existing diabetes did not correlate with kidney dysfunction (in contrast to ET and MF), the reason for this being unclear. Moreover, when correlating MPN-specific therapy to median eGFR, we detected a lower median eGFR in the treated patients compared with WW management in ET and MF but not in PV, suggesting that treatment of PV was beneficial for kidney function. This is in line with previous data showing that treatment of PV improved kidney function and suggests that PV-related non-diabetes factors, such as microcirculatory disturbance and arterial hypertension, play a prominent role in the pathophysiology of CKD in PV [25].

As expected from studies in the general population [47], higher uric acid levels were associated with impaired kidney function in all MPN subtypes, especially in PV and MF, reflecting decreased renal uric acid excretion. However, our study showed correlations of elevated uric acid levels with high cell turnover, even in patients with preserved renal function (Table S5), demonstrating that not only renal dysfunction but also higher cell counts and turnover contribute to higher uric acid levels in MPN patients.

In the overall population, JAK2V617F positivity was not different between eGFR groups (Table S1). However, in patients with the ET subtype, the JAK2V617F mutation occurred more frequently in the eGFR3 group than the other two eGFR groups (Tables S2–S4). Interestingly, recent data show a pathophysiological role of the JAK-STAT pathway in kidney disorders [48,49]. In addition, clinical data suggest an improvement of kidney function with RUX treatment [28,50,51]. The distribution of JAK2V617F positivity in ET and MF was comparable, but MF was associated with more severe CKD and with shorter MPN duration, and thus, other factors may override the impact of JAK2V617F positivity on renal function in MF. In line with these data, Christensen et al. [24] had described a significant decline in eGFR over time in MF patients, which was not present in ET or PV. Therefore, based on our findings and previous reports, a high vigilance regarding kidney function is required in MPN patients, especially those with MF.

As expected, the prevalence of thrombosis was higher in our MPN cohort (29% of all patients) in comparison to general population-based analyses [14,15,16,18], and it was increased in patients with renal dysfunction (Figure 1B). Besides renal dysfunction, relevant risk factors for thrombosis were JAK2V617F positivity, arterial hypertension, hyperlipidemia, and a non-excessive platelet count (Table 3). As most thromboses occur around diagnosis [3] and creatinine measurement was performed after diagnosis in our analysis, it cannot be formally excluded that thrombosis occurred independently from renal dysfunction. However, the fact that JAK2V617F positivity is a known risk factor for thrombosis [1], that CKD was associated with thrombosis in a prior series of PV and ET patients [26], and that the correlation between CKD and thrombosis was most prominent in PV patients (Table S11 and [3,26,52,53]), suggests that rigorous treatment of preventable cardiovascular risk factors may mitigate kidney dysfunction and thrombosis, at least in PV. Only arterial hypertension and non-excessive platelets remained significant upon multivariable analysis. The association between thrombosis and non-excessive platelet counts probably reflects the increased bleeding rate and thus decreased thrombosis rate in patients with platelets over 1000/nL. The association between thrombosis and anti-MPN and antithrombotic therapy is explained by the fact that both therapeutic measures are indicated when a thrombotic event occurs. Prospective data in future research could reveal this confounded association introduced by the cross-sectional nature of the present study. Leukocytosis has been described as an independent risk factor for thrombosis in PV in some studies (meta-analysis in [54]) but not others [55]). In our analysis, we did not assess this risk separately for the MPN subtypes, but leukocytosis was not a risk factor for thrombosis in the univariate or multivariable analysis (Table 3). Contrary to thrombosis, kidney dysfunction was not a risk factor for severe hemorrhage in our cohort (Table 4).

In our cohort, more patients with impaired kidney function received therapy than those with normal kidney function (Table S13), and the fraction of patients aged >60 years was significantly higher in the eGFR3 than the other two eGFR groups (Table S1). In line with these data, median eGFR was significantly lower in ET patients receiving HU and in MF patients receiving HU or RUX treatment compared with those patients managed by WW (Table 5), most likely reflecting their need of pharmacologic treatment because of advanced age rather than a causal role of kidney dysfunction. Previous reports showed that MPN treatment is able to improve CKD in PV but not in ET or MF [24,25]. In the present analysis, no serial measurements of kidney function were available, but the lower frequency of CKD in treated PV patients suggests a positive impact of MPN therapy on kidney function in PV.

An elevated creatinine level was associated with a lower survival rate in ET but not PV or MF (Figure 2A–C), suggesting that, in PV and MF, other risk factors are more relevant for survival. This was particularly relevant since the median age of ET patients was only 54 years at diagnosis. Time between diagnosis and creatinine testing was 5, 6, and 3 years, respectively, in ET, PV, and MF patients (Table 1).

To our knowledge, this is the largest MPN cohort analyzed for renal dysfunction to date, and, importantly, it reflects real world data from current practice. Nevertheless, our study has some limitations: (a) We have only one measurement of serum creatinine; the other parameters were extracted from patients’ records and only the life-time prevalence of thrombosis was documented. Therefore, no rigorous cause-effect relationships between MPN, CKD, and thrombosis can be demonstrated statistically. However, the assumed relationships between MPN, CKD, and thrombosis in our study are supported by prior research relevant in the context (see above). Nevertheless, paired measurements of creatinine and related parameters at predefined time points, i.e., before and at the time of diagnosis, during FU, and the date of thrombosis, should be analyzed in future prospective studies. (b) Furthermore, additional parameters for kidney dysfunction such as albumin, serum cystatine C [56], and additional inflammatory biomarkers in blood and urine may be better indicators of renal pathophysiology, since creatinine is also influenced by plasma volume and muscle mass. Adding these factors and more follow-up data would allow us to develop a time-dependent risk model comprising MPN duration, thrombosis, the course of CKD, and the effects of anti-MPN therapy. (c) Finally, prospective trials will be needed to establish evidence for the benefit of vigilance for renal dysfunction and early intervention to decrease morbidity in MPN patients.

## 5. Conclusions

Our large registry-based multicenter study revealed an increased prevalence of kidney dysfunction in MPN patients compared with the general population and, in addition, we demonstrated a higher proportion of thromboses in patients with more severe kidney dysfunction. It was shown that MF has a higher impact on kidney function compared with ET or PV. The lower frequency of CKD in PV patients receiving MPN treatment may imply a beneficial impact of MPN treatment on kidney function. In conclusion, our results suggest that MPN patients with renal dysfunction may require closer monitoring and, possibly, earlier thromboprophylaxis and MPN-directed treatment. Nevertheless, future research should focus on the acquisition of longitudinal data, which is required to confirm the findings of the present study.

## Figures and Tables

**Table 1:** Patient characteristics of the total cohort and by MPN subtypes.

Parameters (Available *n*)	All MPN	ET	PV	MF	*P*	
					Overall	Between MPN Subtypes
Diagnosis (*n*)	1420	546	478	396	-	-
**Fractional Parameters; %**						
MPN subtype (*n* = 1420)	100	38.5	33.7	27.9	-	-
Male gender (*n* = 1420)	48.9	41.6	49.8	57.8	**<0.0001**	EvP
JAK2V617F mutation pos (*n* = 1315)	73.3	63.2	92.3	63.5	**<0.0001**	EvP/PvM
HU-treated (*n* = 1215) *^,†^	39.6	35.9	53.4	28.9	**<0.0001**	EvP/PvM
RUX-treated (*n* = 1215) *^,‡^	18.8	4.7	16.7	40.1	**<0.0001**	EvP/EvM/PvM
Other MPN treatment (*n* = 1215) ^§^	21.3	30.2	12.4	19.5	**<0.0001**	EvP/EvM/PvM
ASA-treated (*n* = 1252)	57.7	62.8	66.8	40.0	**<0.0001**	EvM/PvM
P2Y12-antagonist-treated (*n* = 1208) ^||^	5.3	8.1	4.2	2.7	**0.0014**	EvM
Anticoagulant-treated (*n* = 1420) ^¶^	11.6	9.5	15.5	9.6	**0.0061**	EvP/PvM
Kidney function by eGFR subgroup (*n* = 1420)	-	-	-	-	**0.0188**	-
eGFR1 (*n* = 304)	21.4	23.6	21.3	18.4	0.1589	-
eGFR2 (*n* = 799)	56.3	56.4	58.4	53.5	0.3565	-
eGFR3 (*n* = 317)	22.3	20.0	20.3	28.0	**0.0057**	EvM/PvM
Thromboembolism, all (*n* = 1420)	29.2	26.0	33.7	28.0	**0.0223**	EvP
Arterial TE (*n* = 1409)	18.5	17.6	22.2	15.4	**0.0287**	PvM
Venous TE (*n* = 1409)	12.3	9.7	14.2	13.6	0.0581	-
Severe hemorrhage (*n* = 1420)	3.9	3.1	4.2	4.6	0.4841	-
Diabetes mellitus (*n* = 1251)	7.8	6.4	9.5	7.5	0.2130	-
Arterial hypertension	49.0	45.6	62.8	38.6	**<0.0001**	EvP/PvM
Hyperlipidemia	11.6	10.6	17.0	8.8	0.383	-
ECOG-score (*n* = 985) ^#,^ **	-	-	-	-	0.0013	EvM ^††^
ECOG 0 (*n* = 683)	69.3	75.1	70.9	60.1	-	-
ECOG 1 (*n* = 270)	27.4	22.2	27.7	34.0	-	-
ECOG 2 (*n* = 25)	2.5	2.4	1.2	4.2	-	-
ECOG 3 (*n* = 7)	0.7	0.3	0.3	1.8	-	-
**Numeric Parameters; median** **Q1, Q3**						
Age diagnosis, years (*n* = 1416)	57 47, 68	54 43, 65	59 49, 70	60 50, 69	**<0.0001**	EvP/EvM
Age creatinine test, years (*n* = 1420)	62 51, 72	59 48, 70	65 54, 74	63 54, 73	**<0.0001**	EvP/EvM
Age at first MPN treatment, years (*n* = 1215)	60 49, 70	58 45, 67	63 52, 72	60 50, 71	**0.0001**	EvP
MPN duration, months (*n* = 1183)	42 11, 105	46 12, 125	57 14, 130	29 7, 68	**<0.0001**	EvM/PvM
Leukocytes, G/L (*n* = 1408)	8.4 6.1, 11.7	8.2 6.2, 10.3	9.0 6.8, 12.8	7.9 5.4, 12.9	**<0.0001**	EvP/PvM
Platelets, G/L (*n* = 1395)	492 290, 734	678 502, 924	419 278, 604	336 159, 547	**<0.0001**	EvP/EvM/PvM
Creatinine, mg/dL (*n* = 1420)	0.9 0.8, 1.1	0.9 0.8, 1.0	0.90 0.8, 1.1	1.0 0.8, 1.2	**<0.0001**	EvM/PvM
eGFR, mL/min/1.73 m^2^ (*n* = 1420)	74 62, 87	77 64, 89	74 63, 87	72 58, 86	**0.0280**	EvM
Uric acid, mg/dL (*n* = 1012)	5.7 4.5, 6.9	5.2 4.3, 6.3	5.9 4.7, 7.1	6.15 5.0, 7.3	**<0.0001**	EvP/EvM
LDH, U/L (*n* = 1356)	268 15, 395	239 198, 293	258 213, 342	435 279, 698	**<0.0001**	EvP/EvM/PvM
CRP, mg/L (*n* = 356)	1.4 0.6, 4.5	1.1 0.5, 3.8	1.3 0.6, 3.3	2.30 0.9, 5.7	**0.0015**	EvM/PvM

EvM indicates ET vs. MF; EvP, ET vs. PV; and PvM, PV vs. MF. CALR mutation status was registered in 509 pts, with 27.3% of these CALR positive (37.0% in ET, 1.6% in PV, and 33.5% in MF (*p* < 0.0001, EvP/PvM)). * Hydroxyurea. ^†^ Treatment started before creatinine measurement. ^‡^ Ruxolitinib. ^§^ Anagrelide, IMIDs, or interferon. ^||^ Clopidogrel/ticagrelor/prasugrel. ^¶^ Vitamin K-antagonist/dabigatran/rivaroxaban/apixaban/heparin/fondaparinux. ^#^ Eastern Cooperative Oncology Group 0 indicates asymptomatic (fully active, able to carry on all pre-disease activities without restriction), 1 symptomatic but completely ambulatory (restricted in physically strenuous activity but ambulatory and able to carry out work of a light or sedentary nature), 2 symptomatic (<50% in bed during the day, ambulatory and capable of all self-care but unable to carry out any work activities, up and about >50% of waking hours), 3 symptomatic (>50% in bed, but not bedbound, capable of only limited self-care, confined to bed or chair ≥50% of waking hours), 4 bedbound (completely disabled, cannot carry on any self-care, totally confined to bed or chair), 5 death. ** ECOG 4 and 5 were 0% each. ^††^ Significance testing for ECOG 0 vs. 1. Bolded values indicate significant *p* values.

**Table 2:** Risk factors for decreased kidney function among all patients and eGFR groups.

Risk Factors for Decreased Kidney Function	Odds Ratio	95% CI	*P*
Univariate regression (*n* = 1420)			
Diagnosis (*n* = 1420)	-	-	**0.0065**
ET vs. MF (*n* = 942)	**0.674**	**0.525–0.867**	**0.0021**
ET vs. PV (*n* = 1024)	0.926	0.730–1.174	0.5243
MF vs. PV (*n* = 874)	**1.372**	**1.060–1.777**	**0.0160**
JAK2V617F mutation (yes vs. no; *n* = 1315)	1.244	0.981–1.576	0.0711
eGFR2 vs. eGFR1 (*n* = 1030)	1.033	0.764–1.395	0.8347
eGFR3 vs. eGFR1 (*n* = 576)	1.435	0.982–2.097	0.0619
eGFR3 vs. eGFR2 (*n* = 1024)	**1.390**	**1.005–1.922**	**0.0464**
Diabetes mellitus (yes vs. no; *n* = 1251)	**1.815**	**1.215–2.711**	**0.0036**
eGFR2 vs. eGFR1 (*n* = 976)	0.663	0.382–1.153	0.1454
eGFR3 vs. eGFR1 (*n* = 542)	**1.878**	**1.071–3.295**	**0.0279**
eGFR3 vs. eGFR2 (*n* = 984)	**2.830**	**1.763–4.543**	**<0.0001**
Arterial hypertension (yes vs. no; *n* = 956)	**2.419**	**1.879–3.114**	**<0.0001**
eGFR2 vs. eGFR1 (*n* = 739)	**1.405**	**1.016–1.943**	**0.0399**
eGFR3 vs. eGFR1 (*n* = 436)	**3.983**	**2.671–5.939**	**<0.0001**
eGFR3 vs. eGFR2 (*n* = 737)	**2.836**	**2.023–3.976**	**<0.0001**
Hyperlipidemia (yes vs. no; *n* = 181)	**3.570**	**1.445–8.819**	**0.0058**
eGFR2 vs. eGFR1 (*n* = 152)	1.815	0.477–6.905	0.3820
eGFR3 vs. eGFR1 (*n* = 81)	**6.220**	**1.501–25.779**	**0.0118**
eGFR3 vs. eGFR2 (*n* = 129)	**3.429**	**1.207–9.739**	**0.0207**
Leukocytes (>8.4 vs. ≤8.4 G/L; *n* = 1408)	**1.367**	**1.116–1.675**	**0.0025**
eGFR2 vs. eGFR1 (*n* = 1093)	1.023	0.785–1.333	0.8670
eGFR3 vs. eGFR1 (*n* = 619)	**1.624**	**1.181–2.231**	**0.0028**
eGFR3 vs. eGFR2 (*n* = 1104)	**1.587**	**1.218–2.067**	**0.0006**
Platelets (>492 vs. ≤492 G/L; *n* = 1395)	0.832	0.679–1.019	0.0755
eGFR2 vs. eGFR1 (*n* = 1080)	1.078	0.827–1.406	0.5774
eGFR3 vs. eGFR1 (*n* = 618)	0.755	0.550–1.036	0.0814
eGFR3 vs. eGFR2 (*n* = 1092)	**0.700**	**0.538–0.911**	**0.0079**
Uric acid (>5.7 vs. ≤5.7 mg/dL; *n* = 1012)	**2.548**	**1.986–3.268**	**<0.0001**
eGFR2 vs. eGFR1 (*n* = 790)	**1.587**	**1.150–2.192**	**0.0050**
eGFR3 vs. eGFR1 (*n* = 444)	**4.351**	**2.923–6.476**	**<0.0001**
eGFR3 vs. eGFR2 (*n* = 790)	**2.741**	**1.971–3.811**	**<0.0001**
Absolute neutrophil count (>5.48 vs. ≤5.48 G/L; *n* = 1254)	**1.397**	**1.126–1.734**	**0.0024**
eGFR2 vs. eGFR1 (*n* = 981)	1.077	0.814–1.426	0.6019
eGFR3 vs. eGFR1 (*n* = 545)	**1.690**	**1.204–2.373**	**0.0024**
eGFR3 vs. eGFR2 (*n* = 982)	**1.569**	**1.183–2.081**	**0.0018**
Absolute monocyte count (>0.55 vs. ≤0.55 G/L; *n* = 1312)	**1.349**	**1.094–1.664**	**0.0052**
eGFR2 vs. eGFR1 (*n* = 1018)	0.972	0.739–1.278	0.8382
eGFR3 vs. eGFR1 (*n* = 579)	**1.589**	**1.144–2.207**	**0.0058**
eGFR3 vs. eGFR2 (*n* = 1027)	**1.635**	**1.243–2.150**	**0.0004**
LDH (>267.5 vs. ≤267.5 U/L; *n* = 1356)	**1.533**	**1.245–1.887**	**<0.0001**
eGFR2 vs. eGFR1 (*n* = 1056)	1.197	0.913–1.570	0.1940
eGFR3 vs. eGFR1 (*n* = 592)	**1.949**	**1.405–2.703**	**<0.0001**
eGFR3 vs. eGFR2 (*n* = 1064)	**1.628**	**1.241–2.136**	**0.0004**
CRP (>1.4 vs. ≤1.4 mg/L; *n* = 356)	**1.633**	**1.091–2.444**	**0.0172**
eGFR2 vs. eGFR1 (*n* = 273)	0.980	0.579–1.660	0.9411
eGFR3 vs. eGFR1 (*n* = 162)	**2.109**	**1.125–3.953**	**0.0200**
eGFR3 vs. eGFR2 (*n* = 277)	**2.151**	**1.269–3.645**	**0.0044**
MPN duration (>42 vs. ≤42 months; *n* = 1183)	**1.507**	**1.207–1.882**	**0.0003**
eGFR2 vs. eGFR1 (*n* = 904)	**1.482**	**1.101–1.996**	**0.0095**
eGFR3 vs. eGFR1 (*n* = 523)	**1.913**	**1.350–2.710**	**0.0003**
eGFR3 vs. eGFR2 (*n* = 939)	1.290	0.973–1.710	0.0764
MPN therapy (yes vs. WW; *n* = 1215) *	**1.784**	**1.428–2.230**	**<0.0001**
eGFR2 vs. eGFR1 (*n* = 941)	1.267	0.952–1.686	0.1049
eGFR3 vs. eGFR1 (*n* = 535)	**2.514**	**1.762–3.587**	**<0.0001**
eGFR3 vs. eGFR2 (*n* = 939)	**1.985**	**1.470–2.679**	**<0.0001**
HU-treated (yes vs. WW; *n* = 780)	**1.734**	**1.286–2.338**	**0.0003**
eGFR2 vs. eGFR1 (*n* = 639)	**1.639**	**1.099–2.444**	**0.0153**
eGFR3 vs. eGFR1 (*n* = 316)	**2.421**	**1.493–3.926**	**0.0003**
eGFR3 vs. eGFR2 (*n* = 605)	**1.477**	**1.004–2.172**	**0.0475**
RUX-treated (yes vs. WW; *n* = 618)	**2.412**	**1.560–3.728**	**<0.0001**
eGFR2 vs. eGFR1 (*n* = 506)	1.517	0.810–2.842	0.1928
eGFR3 vs. eGFR1 (*n* = 260)	**3.663**	**1.839–7.294**	**0.0002**
eGFR3 vs. eGFR2 (*n* = 470)	**2.413**	**1.446–4.027**	**0.0007**
Other MPN treatment (yes vs. WW; *n* = 865)	**1.737**	**1.331–2.267**	**<0.0001**
eGFR2 vs. eGFR1 (*n* = 682)	1.006	0.714–1.417	0.9734
eGFR3 vs. eGFR1 *(n* = 389)	**2.344**	**1.557–3.527**	**<0.0001**
eGFR3 vs. eGFR2 (*n* = 659)	**2.330**	**1.647–3.297**	**<0.0001**
Antithrombotic therapy (yes vs. no; *n* = 1251)	1.196	0.946–1.511	0.1348
eGFR2 vs. eGFR1 (*n* = 967)	1.296	0.958–1.752	0.0925
eGFR3 vs. eGFR1 (*n* = 548)	1.318	0.918–1.892	0.1314
eGFR3 vs. eGFR2 (*n* = 939)	1.017	0.750–1.380	0.9113
Multiple Regression			
eGFR2 vs. eGFR1 (*n* = 639) *	-	-	-
HU-treated (yes vs. WW)	**1.639**	**1.099–2.444**	**0.0153**
eGFR3 vs. eGFR1 (*n* = 278) ^†,‡^	-	-	-
Arterial hypertension (yes vs. no)	**3.073**	**1.746–5.405**	**<0.0001**
Uric acid (>5.7 vs. ≤ 5.7 mg/dL)	**4.918**	**2.787–8.677**	**<0.0001**
HU-treated (yes vs. WW)	**3.509**	**1.630–7.557**	**0.0013**
RUX-treated (yes vs. WW)	**5.416**	**1.927–15.223**	**0.0014**
Other MPN treatment (yes vs. WW)	**2.477**	**1.277–4.803**	**0.0073**
eGFR3 vs. eGFR2 (*n* = 589) ^§^	-	-	-
Arterial hypertension (yes vs. no)	**2.004**	**1.440–2.789**	**<0.0001**
Uric acid (>5.7 vs. ≤5.7 mg/dL)	**2.254**	**1.617–3.140**	**<0.0001**
LDH (>267.5 vs. ≤267.5 U/L)	**1.448**	**1.036–2.023**	**0.0301**
HU-treated (yes vs. WW)	**1.797**	**1.162–2.780**	**0.0085**
Other MPN treatment (yes vs. WW)	**1.645**	**1.095–2.471**	**0.0165**

* Initial model for eGFR2 vs. eGFR1: arterial hypertension, uric acid, MPN duration, and HU-treated. ^†^ Initial model for eGFR3 vs. eGFR1: diabetes mellitus, arterial hypertension, leukocytes, and uric acid; neutrophils, monocytes, LDH, CRP, MPN duration, HU-treated, RUX-treated, and other MPN treatment. ^‡^ Hyperlipidemia (*n* = 69) was not assessed in multiple regression analysis because of the low number of patients. ^§^ Initial model for eGFR3 vs. eGFR2: JAK2V617F mutation, diabetes mellitus, arterial hypertension, leukocytes, platelets, and uric acid; neutrophils, monocytes, LDH, CRP, HU-treated, RUX-treated, and other MPN treatment. Bolded values indicate significant odds ratios and significant *p* values.

**Table 3:** Risk factors for thrombosis (*n* = 414) among all patients and eGFR groups.

Risk Factors for Thromboembolic Event	Odds Ratio	95% CI	*P*
Univariate regression (*n* = 1420)			
eGFR group (*n* = 1420)	-	-	**0.0145**
eGFR3 vs. eGFR2 (*n* = 1116)	**1.449 **	**1.098–1.912 **	**0.0088 **
eGFR3 vs. eGFR1 (*n* = 621)	**1.551 **	**1.100–2.187 **	**0.0123 **
eGFR2 vs. eGFR1 (*n* = 1103)	1.071	0.794–1.443	0.6544
ET (*n* = 546)	-	-	0.3744
eGFR3 vs. eGFR2 (*n* = 417)	1.408	0.870–2.280	0.1634
eGFR3 vs. eGFR1 (*n* = 238)	1.319	0.749–2.323	0.3381
eGFR2 vs. eGFR1 (*n* = 437)	0.936	0.583–1.503	0.7856
PV (*n* = 478)	-	-	**0.0258 **
eGFR3 vs. eGFR2 (*n* = 376)	**1.863**	**1.160–2.992**	**0.0100**
eGFR3 vs. eGFR1 (*n* = 199)	**1.901**	**1.063–3.400**	**0.0302**
eGFR2 vs. eGFR1 (*n* = 381)	1.021	0.624–1.670	0.9355
MF (*n* = 396)	-	-	0.3649
eGFR3 vs. eGFR2 (*n* = 323)	1.167	0.708–1.923	0.5453
eGFR3 vs. eGFR1 (*n* = 184)	1.640	0.828–3.251	0.1561
eGFR2 vs. eGFR1 (*n* = 285)	1.406	0.749–2.640	0.2890
Diagnosis (*n* = 1420)	-	-	**0.0227 **
ET vs. PV (*n* = 1024)	**0.692**	**0.529–0.906**	**0.0074**
ET vs. MF (*n* = 942)	0.902	0.675–1.207	0.4894
MF vs. PV (*n* = 874)	0.767	0.574–1.025	0.0727
Sex (female vs. male; *n* = 1420)	0.819	0.651–1.029	0.0869
Age at diagnosis (>60 vs. ≤60 years; *n* = 1416)	1.219	0.968–1.535	0.0924
Age at creatinine test (>60 vs. ≤60 years; *n* = 1420)	1.190	0.945–1.500	0.1397
JAK2V617F mutation (yes vs. no; *n* = 1315)	**1.632**	**1.227–2.169**	**0.0007**
Diabetes mellitus (yes vs. no; *n* = 1251)	1.245	0.803–1.930	0.3279
Arterial hypertension (yes vs. no; *n* = 956)	**1.838**	**1.398–2.418**	**<0.0001**
Hyperlipidemia (yes vs. no; *n* = 181)	**2.936**	**1.149–7.503**	**0.0245**
Leukocytes (>8.4 vs. ≤8.4 G/L; *n* = 1408)	0.850	0.676–1.070	0.1668
Platelets (>492 vs. ≤492 G/L; *n* = 1395)	**0.633**	**0.502–0.799**	**0.0001**
Uric acid (>5.7 vs. ≤5.7 mg/dL; *n* = 1012)	1.166	0.888–1.531	0.2685
Absolute neutrophil count (>5.48 vs. ≤5.48 G/L; *n* = 1254)	0.807	0.632–1.030	0.0843
Absolute monocyte count (>0.55 vs. ≤0.55 G/L; *n* = 1312)	1.041	0.821–1.322	0.7385
LDH (>267.5 vs. ≤267.5 U/L; *n* = 1356)	1.246	0.986–1.574	0.0653
CRP (>1.4 vs. ≤1.4 mg/L; *n* = 356)	1.194	0.768–1.855	0.4308
MPN therapy (yes vs. WW; *n* = 1215)	**1.516**	**1.183–1.944**	**0.0010**
HU-treated (yes vs. WW; *n* = 780)	**1.592**	**1.156–2.191**	**0.0043**
RUX-treated (yes vs. WW; *n* = 618)	1.164	0.718–1.885	0.5382
Other MPN treatment (yes vs. WW; *n* = 865)	**1.567**	**1.169–2.102**	**0.0027**
Antithrombotic therapy (yes vs. no; *n* = 1251)	**4.407**	**3.178–6.111**	**<0.0001**
ASA (yes vs. no; *n* = 1029)	**2.622**	**1.862–3.694**	**<0.0001**
P2Y12-antagonists (yes vs. no; *n* = 405)	**12.999**	**5.752–29.387**	**<0.0001**
Anticoagulant-treated (yes vs. no; *n* = 497)	**22.867**	**13.583–38.498**	**<0.0001**
Multiple regression (*n* = 911) *^,†^			
Arterial hypertension (yes vs. no)	**1.800**	**1.349–2.401**	**0.0117**
Platelets (>492 vs. ≤492 G/L)	**0.699**	**0.524–0.931**	**0.0068**
ASA (yes vs. no)	**2.885**	**1.905–4.371**	**<0.0001**
P2Y12-antagonists (yes vs. no)	**12.957**	**5.040–33.311**	**<0.0001**
Anticoagulant-treated (yes vs. no)	**20.284**	**11.175–36.815**	**<0.0001**
By Diagnosis			
ET (*n* = 347)	-	-	-
Arterial hypertension (yes vs. no)	**1.913**	**1.099–3.330**	**0.0217**
RUX-treated (yes vs. WW)	**10.379**	**1.572–68.513**	**0.0151**
Other MPN treatment (yes vs. WW)	**2.013**	**1.108–3.656**	**0.0216**
ASA (yes vs. no)	**2.094**	**1.003 -4.374**	**0.0491**
P2Y12-antagonists (yes vs. no)	**12.235**	**3.362–44.524**	**0.0001**
Anticoagulant-treated (yes vs. no)	**29.068**	**9.167–92.171**	**<0.0001**
PV (*n* = 377)	-	-	-
Other MPN treatment (yes vs. WW)	**2.270**	**1.250–4.120**	**0.0071**
Anticoagulant-treated (yes vs. no)	**12.732**	**5.271–30.756**	**<0.0001**
MF (*n* = 267)	-	-	-
Arterial hypertension (yes vs. no)	**1.960**	**1.067–3.601**	**0.0301**
ASA (yes vs. no)	**5.546**	**2.837–10.843**	**<0.0001**
Anticoagulant-treated (yes vs no)	**22.606**	**7.167–71.304**	**<0.0001**

* Initial model includes eGFR group, diagnosis, JAK2V617F mutation, arterial hypertension, platelets, MPN therapy, antithrombotic therapy. ^†^ Hyperlipidemia (*n* = 102) was not assessed by multiple regression analysis because of the low number of patients. Bolded values indicate significant odds ratios and significant *p* values.

**Table 4:** Risk factors for severe bleeding (*n* = 55) among all patients and eGFR groups.

Risk Factors for Bleeding Event	ODDS RATIO	95% CI	*P*
Univariate regression (*n* = 1420)			
eGFR group (*n* = 1420)	-	-	0.6469
eGFR3 vs. eGFR2 (*n* = 1116)	0.807	0.391–1.666	0.5624
eGFR3 vs. eGFR1 (*n* = 621)	0.675	0.295–1.543	0.3513
eGFR2 vs. eGFR1 (*n* = 1103)	0.836	0.438–1.594	0.5865
ET (*n* = 546)	-	-	0.2269
eGFR3 vs. eGFR2 (*n* = 417)	0.701	0.147–3.353	0.6563
eGFR3 vs. eGFR1 (*n* = 238)	0.326	0.066–1.602	0.1675
eGFR2 vs. eGFR1 (*n* = 437)	0.465	0.165–1.309	0.1471
PV (*n* = 478)	-	-	0.5219
eGFR3 vs. eGFR2 (*n* = 376)	0.431	0.095–1.944	0.2734
eGFR3 vs. eGFR1 (*n* = 199)	0.408	0.077–2.157	0.2916
eGFR2 vs. eGFR1 (*n* = 381)	0.948	0.329–2.729	0.9213
MF (*n* = 396)	-	-	0.6940
eGFR3 vs. eGFR2 (*n* = 323)	1.154	0.408–3.263	0.7867
eGFR3 vs. eGFR1 (*n* = 184)	2.029	0.398–10.337	0.3946
eGFR2 vs. eGFR1 (*n* = 285)	1.757	0.376–8.215	0.4736
Diagnosis (*n* = 1420)	-	-	0.4877
ET vs. PV (*n* = 1024)	0.736	0.381–1.422	0.3618
ET vs. MF (*n* = 942)	0.675	0.343–1.327	0.2543
MF vs. PV (*n* = 874)	1.091	0.569–2.091	0.7941
Sex (female vs. male; *n* = 1420)	**0.533**	**0.305–0.933**	**0.0277**
Age at diagnosis (>60 vs. ≤60 years; *n* = 1416)	0.756	0.432–1.323	0.0924
Age at creatinine test (>60 vs. ≤60 years; *n* = 1420)	0.784	0.457–1.344	0.3758
JAK2V617F mutation (yes vs. no; *n* = 1315)	1.759	0.813–3.809	0.1517
Diabetes mellitus (yes vs. no; *n* = 1251)	1.439	0.556–3.725	0.4534
Arterial hypertension (yes vs. no; *n* = 956)	1.302	0.678–2.499	0.4284
Hyperlipoproteinemia (yes vs. no; *n* = 181)	2.661	0.779–9.086	0.1182
Leukocytes (>8.4 vs. ≤8.4 G/L; *n* = 1408)	1.543	0.890–2.674	0.1223
Platelets (>492 vs. ≤492 G/L; *n* = 1395)	0.560	0.320–0.979	**0.0421**
Uric acid (>5.7 vs. ≤5.7 mg/dL; *n* = 1012)	1.685	0.869–3.269	0.1225
Absolute neutrophil count (>5.48 vs. ≤5.48 G/L; *n* = 1254)	1.697	0.963–2.992	0.0673
Absolute monocyte count (>0.55 vs. ≤0.55 G/L; *n* = 1312)	0.980	0.566–1.698	0.9424
LDH (>267.5 vs. ≤267.5 U/L; *n* = 1356)	3.292	1.748–6.201	**0.0002**
CRP (>1.4 vs. ≤1.4 mg/L; *n* = 356)	0.868	0.351–2.150	0.7603
MPN therapy (yes vs. WW; *n* = 1215)	1.694	0.927 -3.095	0.0865
Antithrombotic therapy (yes vs. WW; *n* = 1251)	0.903	0.500–1.628	0.7333
Multiple regression (*n* = 1335) *			
LDH (>267.5 vs. ≤267.5 U/L; *n* = 1356)	3.292	1.748–6.201	**0.0002**

* Initial model including sex, platelets, and LDH. Bolded values indicate significant odds ratios and significant *p* values.

**Table 5:** Median eGFR in patients treated with HU, RUX, or anagrelide/IMIDs/interferon.

Sample (*n* = 1215)	Therapy	*n* (%)	eGFR mL/min/1.73 m^2^			*P*	
			Median	Q1	Q3	Overall	Pairwise *
All patients (*n* = 1215)						**<0.0001**	
	HU only	256 (21.07)	71.90	60.58	84.39		**0.0005**
	RUX only	94 (7.74)	68.56	56.23	84.48		**0.0002**
	Other	341 (28.07)	72.77	57.40	85.95		
	Multiple therapies	250 (20.58)	71.00	54.41	84.62		**0.0021**
	WW	524 (43.13)	77.81	66.48	90.23		
ET (*n* = 471)						**0.0013**	
	HU only	88 (18.68)	72.43	63.26	86.72		**0.0168**
	RUX only	5 (1.06)	60.79	58.00	61.52		0.0588
	Other	148 (31.42)	76.70	57.48	89.24		
	Multiple therapies	88 (18.68)	74.37	55.71	89.24		>0.9999
	WW	230 (48.83)	78.55	69.47	91.42		
PV (*n* = 395)						0.6865	
	HU only	135 (34.18)	72.88	60.43	84.79		0.3516
	RUX only	11 (2.78)	72.21	55.75	81.59		0.8951
	Other	90 (22.78)	72.63	59.32	89.69		
	Multiple therapies	77 (19.49)	70.11	58.69	83.20		0.3453
	WW	159 (40.25)	74.36	63.05	87.20		
MF (*n* = 349)						**0.0073**	
	HU only	33 (9.46)	68.90	58.61	73.45		**0.0488**
	RUX only	78 (22.35)	68.85	56.23	84.69		**0.0188**
	Other	103 (29.51)	69.74	53.46	81.02		
	Multiple therapies	85 (24.36)	69.74	52.67	80.35		0.2208
	WW	135 (38.68)	76.29	62.46	91.20		

* Therapy compared to WW. Bolded values indicate significant *p* values.

**Table 6:** Median eGFR in patients treated with ASA, P2Y12-antagonists, or anticoagulation therapy.

Sample (*n* = 1251)	Therapy	n (%)	eGFR mL/min/1.73 m^2^			*P*	
			Median	Q1	Q3	Overall	Pairwise *
All patients (*n* = 1251)						0.1462	
	ASA	654 (52.28)	75.00	63.18	86.25		0.9909
	P2Y12-antagonists	30 (2.40)	74.50	59.14	83.25		0.8892
	Anticoagulant-treated	122 (9.75)	70.65	55.36	86.30		0.1092
	No	375 (29.98)	75.23	62.37	89.19		
ET (*n* = 483)						**0.0234**	
	ASA	272 (56.31)	76.78	64.87	88.05		0.1026
	P2Y12-antagonists	19 (3.93)	74.47	52.69	85.74		0.1713
	Anticoagulant-treated	34 (7.04)	78.82	54.61	89.15		0.5919
	No	124 (25.67)	79.09	67.91	94.05		
PV (*n* = 415)						0.3127	
	ASA	254 (61.20)	74.97	63.44	86.25		0.7041
	P2Y12-antagonists	7 (1.69)	81.36	66.91	88.84		0.7107
	Anticoagulant-treated	59 (14.22)	70.81	57.15	89.84		>0.9999
	No	73 (17.59)	70.50	59.60	83.97		
MF (*n* = 349)						0.0849	
	ASA	128 (36.26)	71.33	59.84	81.12		0.2907
	P2Y12-antagonists	4 (1.13)	65.46	49.50	76.41		0.8580
	Anticoagulant-treated	29 (8.22)	62.06	52.36	83.70		0.2262
	no	178 (50.42)	73.16	59.20	87.80		

* Therapy compared to no therapy. Bolded values indicate significant *p* values.

## Data Availability

The datasets used and/or analyzed during the current study are available from the corresponding author on reasonable request.

## Supplementary Materials

The following are available online at https://www.mdpi.com/article/10.3390/cancers13164086/s1, Table S1: Patient characteristics of the total cohort and by eGFR groups; Table S2: Patient characteristics by eGFR groups in ET patients; Table S3: Patient characteristics by eGFR groups in PV patients; Table S4: Patient characteristics by eGFR groups in MF patients; Table S5: Correlations between uric acid, leukocytes, LDH, and platelets; Table S6: Median MPN duration across eGFR groups; Table S7: MPN duration of >42 months versus ≤ 42 months across eGFR groups; Table S8: Median MPN duration across creatinine groups; Table S9: MPN duration of >42 months versus ≤42 months across creatinine groups; Table S10: Risk factors for MPN duration longer than 42 months; Table S11: Frequencies of thromboses across eGFR groups by risk factor; Table S12: Distribution of arterial and venous thromboembolism across eGFR groups; Table S13: Frequency of HU, RUX, anagrelide/IMIDs/interferon, and no therapy across eGFR groups; Table S14: Frequency of ASA, P2Y12-antagonists, or anticoagulation therapy across eGFR groups.

## Abbreviations

ADL	Activities of daily living
ASA	Acetylsalicylic acid
CI	Confidence interval
CKD	Chronic kidney disease
CRP	C-reactive protein
ECOG PS	Eastern Cooperative Oncology Group Performance Status
eGFR	estimated glomerular filtration rate
ET	Essential thrombocythemia
FU	Follow-up
GSG-MPN bioregistry	Myeloproliferative neoplasms bioregistry study of the German Study Group for myeloproliferative neoplasms
HR	Hazard ratio
HU	Hydroxyurea
IMIDs	Immunomodulatory drugs
IWG-MRT	International Working Group-Myeloproliferative Neoplasms Research and Treatment
KDIGO	Kidney Disease Improving Global Outcomes
LDH	Lactate dehydrogenase
MDRD	Modification of Diet in Renal Disease
MF	Myelofibrosis
MPN	Myeloproliferative neoplasms
OR	Odds ratio
PMF	Primary myelofibrosis
PV	Polycythemia vera
Q1	First quartile
Q3	Third quartile
RUX	Ruxolitinib
Scr	Serum creatinine
WHO	World Health Organization
WW	Watch-and-wait

## References

[1] Barbui T., Finazzi G., Falanga A. (2013). Myeloproliferative neoplasms and thrombosis. Blood.

[2] Hultcrantz M., Björkholm M., Dickman P.W., Landgren O., Derolf Å.R., Kristinsson S.Y., Andersson T.M.L. (2018). Risk for Arterial and Venous Thrombosis in Patients With Myeloproliferative Neoplasms: A Population-Based Cohort Study. Ann. Intern. Med..

[3] Kaifie A., Kirschner M., Wolf D., Maintz C., Hänel M., Gattermann N., Gökkurt E., Platzbecker U., Hollburg W., Göthert J.R. (2016). Bleeding, thrombosis, and anticoagulation in myeloproliferative neoplasms (MPN): Analysis from the German SAL-MPN-registry. J. Hematol. Oncol..

[4] McMahon B., Stein B.L. (2013). Thrombotic and bleeding complications in classical myeloproliferative neoplasms. Semin. Thromb. Hemost..

[5] Reikvam H., Tiu R.V. (2012). Venous thromboembolism in patients with essential thrombocythemia and polycythemia vera. Leukemia.

[6] Price G.L., Davis K.L., Karve S., Pohl G., Walgren R.A. (2014). Survival patterns in United States (US) medicare enrollees with non-CML myeloproliferative neoplasms (MPN). PLoS ONE.

[7] Campbell P.J., MacLean C., Beer P.A., Buck G., Wheatley K., Kiladjian J.-J., Forsyth C., Harrison C.N., Green A. (2012). Correlation of blood counts with vascular complications in essential thrombocythemia: Analysis of the prospective PT1 cohort. Blood.

[8] Carobbio A., Thiele J., Passamonti F., Rumi E., Ruggeri M., Rodeghiero F., Randi M.L., Bertozzi I., Vannucchi A.M., Antonioli E. (2011). Risk factors for arterial and venous thrombosis in WHO-defined essential thrombocythemia: An international study of 891 patients. Blood.

[9] Griesshammer M., Kiladjian J.-J., Besses C. (2019). Thromboembolic events in polycythemia vera. Ann. Hematol..

[10] Landolfi R., Di Gennaro L., Barbui T., De Stefano V., Finazzi G., Marfisi R., Tognoni G., Marchioli R., European Collaboration on Low-Dose Aspirin in Polycythemia Vera (ECLAP) (2007). Leukocytosis as a major thrombotic risk factor in patients with polycythemia vera. Blood.

[11] Vannucchi A.M., Guglielmelli P. (2013). JAK2 mutation-related disease and thrombosis. Semin. Thromb. Hemost..

[12] Zhang Y., Zhou Y., Wang Y., Teng G., Li D., Wang Y., Du C., Chen Y., Zhang H., Li Y. (2020). Thrombosis among 1537 patients with JAK2(V617F) -mutated myeloproliferative neoplasms: Risk factors and development of a predictive model. Cancer Med..

[13] Keith D.S., Nichols G.A., Gullion C.M., Brown J.B., Smith D.H. (2004). Longitudinal follow-up and outcomes among a population with chronic kidney disease in a large managed care organization. Arch. Intern. Med..

[14] Liu M., Li X.-C., Lu L., Cao Y., Sun R.-R., Chen S., Zhang P.-Y. (2014). Cardiovascular disease and its relationship with chronic kidney disease. Eur. Rev. Med. Pharmacol. Sci..

[15] Mathew R.O., Bangalore S., Lavelle M.P., Pellikka P.A., Sidhu M.S., Boden W.E., Asif A. (2017). Diagnosis and management of atherosclerotic cardiovascular disease in chronic kidney disease: A review. Kidney Int..

[16] Provenzano M., Coppolino G., De Nicola L., Serra R., Garofalo C., Andreucci M., Bolignano D. (2019). Unraveling Cardiovascular Risk in Renal Patients: A New Take on Old Tale. Front. Cell Dev. Biol..

[17] Tonelli M., Wiebe N., Culleton B., House A., Rabbat C., Fok M., McAlister F., Garg A.X. (2006). Chronic Kidney Disease and Mortality Risk: A Systematic Review. J. Am. Soc. Nephrol..

[18] Hemmelgarn B.R., Manns B.J., Lloyd A., James M.T., Klarenbach S., Quinn R.R., Wiebe N., Tonelli M., Alberta Kidney Disease Network (2010). Relation between kidney function, proteinuria, and adverse outcomes. JAMA.

[19] Ocak G., Lijfering W.M., Verduijn M., Dekker F., Rosendaal F.R., Cannegieter S.C., Vossen C.Y. (2013). Risk of venous thrombosis in patients with chronic kidney disease: Identification of high-risk groups. J. Thromb. Haemost..

[20] Menon V., Greene T., Wang X., Pereira A.A., Marcovina S.M., Beck G.J., Kusek J.W., Collins A.J., Levey A.S., Sarnak M.J. (2005). C-reactive protein and albumin as predictors of all-cause and cardiovascular mortality in chronic kidney disease. Kidney Int..

[21] Asaba K., Tojo A., Onozato M.L., Mimura N., Kido M., Goto A., Endo H., Fujita T. (2003). Fibrillary glomerulonephritis associated with essential thrombocytosis. Clin. Exp. Nephrol..

[22] Okuyama S., Hamai K., Fujishima M., Ohtani H., Komatsuda A., Sawada K., Wakui H. (2007). Focal segmental glomerulosclerosis associated with polycythemia vera: Report of a case and review of the literature. Clin. Nephrol..

[23] Rahimian S., Johnson T., Herb R. (2019). A Case of Essential Thrombocythemia and IgA Nephropathy with Literature Review of the Concurrence. Case Rep. Oncol. Med..

[24] Christensen A.S., Møller J.B., Hasselbalch H.C. (2014). Chronic kidney disease in patients with the Philadelphia-negative chronic myeloproliferative neoplasms. Leuk. Res..

[25] Baek S.-W., Moon J.Y., Ryu H., Choi Y.-S., Song I.-C., Lee H.-J., Yun H.J., Kim S., Jo D.-Y. (2018). Chronic kidney disease in the BCR-ABL1-negative myeloproliferative neoplasm: A single-center retrospective study. Korean J. Intern. Med..

[26] Krečak I., Holik H., Martina M.P., Zekanović I., Coha B., Gverić-Krečak V. (2020). Chronic kidney disease could be a risk factor for thrombosis in essential thrombocythemia and polycythemia vera. Int. J. Hematol..

[27] Lucijanic M., Galusic D., Krecak I., Sedinic M., Holik H., Perisa V., Peric M.M., Zekanovic I., Stoos-Veic T., Kusec R. (2020). Reduced renal function strongly affects survival and thrombosis in patients with myelofibrosis. Ann. Hematol..

[28] Strati P., Abdelrahim M., Selamet U., Page V.D., Pierce S.A., Verstovsek S., Abudayyeh A. (2019). Ruxolitinib therapy is associated with improved renal function in patients with primary myelofibrosis. Ann. Hematol..

[29] Fukuda Y., Araki M., Yamamoto K., Morishita S., Inano T., Misawa K., Ochiai T., Edahiro Y., Imai M., Yasuda H. (2019). Evidence for prevention of renal dysfunction associated with primary myelofibrosis by cytoreductive therapy. Haematologica.

[30] Tefferi A., Vardiman J.W. (2008). Classification and diagnosis of myeloproliferative neoplasms: The 2008 World Health Organization criteria and point-of-care diagnostic algorithms. Leukemia.

[31] Levey A.S., Stevens L.A., Schmid C.H., Zhang Y.L., Castro A.F., Feldman H.I., Kusek J.W., Eggers P., Lente F.V., Greene T. (2009). A new equation to estimate glomerular filtration rate. Ann. Intern. Med..

[32] Levin A., Stevens P.E. (2014). Summary of KDIGO 2012 CKD Guideline: Behind the scenes, need for guidance, and a framework for moving forward. Kidney Int..

[33] Bar C., Huber N., Beier F., Blasco M.A. (2015). Therapeutic effect of androgen therapy in a mouse model of aplastic anemia produced by short telomeres. Haematologica.

[34] Passamonti F., Thiele J., Girodon F., Rumi E., Carobbio A., Gisslinger H., Kvasnicka H.M., Ruggeri M., Randi M.L., Gangat N. (2012). A prognostic model to predict survival in 867 World Health Organization-defined essential thrombocythemia at diagnosis: A study by the International Working Group on Myelofibrosis Research and Treatment. Blood.

[35] Rumi E., Pietra D., Ferretti V., Klampfl T., Harutyunyan A.S., Milosevic J.D., Them N.C.C., Berg T., Elena C., Casetti I.C. (2014). JAK2 or CALR mutation status defines subtypes of essential thrombocythemia with substantially different clinical course and outcomes. Blood.

[36] Arellano-Rodrigo E., Alvarez-Larran A., Reverter J.C., Colomer D., Villamor N., Bellosillo B., Cervantes F. (2009). Platelet turnover, coagulation factors, and soluble markers of platelet and endothelial activation in essential thrombocythemia: Relationship with thrombosis occurrence and JAK2 V617F allele burden. Am. J. Hematol..

[37] Belotti A., Elli E., Speranza T., Lanzi E., Pioltelli P., Pogliani E. (2012). Circulating endothelial cells and endothelial activation in essential thrombocythemia: Results from CD146+ immunomagnetic enrichment—Flow cytometry and soluble E-selectin detection. Am. J. Hematol..

[38] Hauschner H., Horev M.B., Misgav M., Nagar M., Seligsohn U., Rosenberg N., Koren-Michowitz M. (2019). Platelets from Calreticulin mutated essential thrombocythemia patients are less reactive than JAK2 V617F mutated platelets. Am. J. Hematol..

[39] Yamagata K., Ishida K., Sairenchi T., Takahashi H., Ohba S., Shiigai T., Narita M., Koyama A. (2007). Risk factors for chronic kidney disease in a community-based population: A 10-year follow-up study. Kidney Int..

[40] Coresh J., Astor B.C., Greene T., Eknoyan G., Levey A.S. (2003). Prevalence of chronic kidney disease and decreased kidney function in the adult US population: Third National Health and Nutrition Examination Survey. Am. J. Kidney Dis..

[41] Rifkin D.E., Shlipak M.G., Katz R., Fried L.F., Siscovick D., Chonchol M., Newman A.B., Sarnak M.J. (2008). Rapid kidney function decline and mortality risk in older adults. Arch. Intern. Med..

[42] Hapca S., Siddiqui M.K., Kwan R.S., Lim M., Matthew S., Doney A.S., Pearson E., Palmer C., Bell S., BEAt-DKD Consortium (2020). The Relationship between AKI and CKD in Patients with Type 2 Diabetes: An Observational Cohort Study. J. Am. Soc. Nephrol. JASN.

[43] Patschan D., Muller G.A. (2016). Acute Kidney Injury in Diabetes Mellitus. Int. J. Nephrol..

[44] Skov V., Larsen T.S., Thomassen M., Riley C.H., Jensen M.K., Bjerrum O.W., Kruse T.A., Hasselbalch H. (2012). Molecular profiling of peripheral blood cells from patients with polycythemia vera and related neoplasms: Identification of deregulated genes of significance for inflammation and immune surveillance. Leuk. Res..

[45] Kato S., Chmielewski M., Honda H., Pecoits-Filho R., Matsuo S., Yuzawa Y., Tranaeus A., Stenvinkel P., Lindholm B. (2008). Aspects of immune dysfunction in end-stage renal disease. Clin. J. Am. Soc. Nephrol..

[46] Bazeley J., Bieber B., Li Y., Morgenstern H., De Sequera P., Combe C., Yamamoto H., Gallagher M., Port F.K., Robinson B.M. (2011). C-reactive protein and prediction of 1-year mortality in prevalent hemodialysis patients. Clin. J. Am. Soc. Nephrol..

[47] Obermayr R.P., Temml C., Gutjahr G., Knechtelsdorfer M., Oberbauer R., Klauser-Braun R. (2008). Elevated uric acid increases the risk for kidney disease. J. Am. Soc. Nephrol..

[48] Brosius F.C., He J.C. (2015). JAK inhibition and progressive kidney disease. Curr. Opin. Nephrol. Hypertens..

[49] Brosius F.C., Tuttle K.R., Kretzler M. (2016). JAK inhibition in the treatment of diabetic kidney disease. Diabetologia.

[50] Tabarroki A., Visconte V., Rogers H.J., Sekeres M.A., Samaras C., Lichtin A., Duong H.K., Englehaupt R., Cinalli T., Dodd K. (2014). Clinical experiences with ruxolitinib in symptomatic patients with myeloproliferative neoplasm with chronic kidney disease. Leuk. Lymphoma..

[51] Strati P., Glass W.F., Abdelrahim M., Selamet U., Tchakarov A., Workeneh B., Verstovsek S., Abudayyeh A. (2019). Renal complications of primary myelofibrosis. Leuk. Lymphoma.

[52] Elliott M.A., Tefferi A. (2005). Thrombosis and haemorrhage in polycythaemia vera and essential thrombocythaemia. Br. J. Haematol..

[53] Kreher S., Ochsenreither S., Trappe R., Pabinger I., Bergmann F., Petrides P.E., Koschmieder S., Matzdorff A., Tiede A., Griesshammer M. (2014). Prophylaxis and management of venous thromboembolism in patients with myeloproliferative neoplasms: Consensus statement of the Haemostasis Working Party of the German Society of Hematology and Oncology (DGHO), the Austrian Society of Hematology and Oncology (OGHO) and Society of Thrombosis and Haemostasis Research (GTH e.V.). Ann. Hematol..

[54] Carobbio A., Ferrari A., Masciulli A., Ghirardi A., Barosi G., Barbui T. (2019). Leukocytosis and thrombosis in essential thrombocythemia and polycythemia vera: A systematic review and meta-analysis. Blood Adv..

[55] Ronner L., Podoltsev N., Gotlib J., Heaney M.L., Kuykendall A.T., O’Connell C., Shammo J., Fleischman A.G., Scherber R.M., Mesa R. (2020). Persistent leukocytosis in polycythemia vera is associated with disease evolution but not thrombosis. Blood.

[56] Madero M., Sarnak M.J., Stevens L.A. (2006). Serum cystatin C as a marker of glomerular filtration rate. Curr. Opin. Nephrol. Hypertens..

