# Supplementary material for: Kidney Dysfunction Is Associated with Thrombosis and Disease Severity in Myeloproliferative Neoplasms: Implications from the German Study Group for MPN Bioregistry
Source: Cancers (Basel). 2021 Aug 13;13(16):4086. doi: 10.3390/cancers13164086 (PMC8393882; doi:10.3390/cancers13164086)
Supplement: Supplementary file 1 [file cancers-13-04086-s001.zip › cancers-1281372-supplementary.pdf]

cancers

MDPI

# Supplementary Materials: Kidney Dysfunction Is Associated with Thrombosis and Dis-ease Severity in Myeloproliferative Neoplasms: Implications from the German Study Group for MPN Bioregistry

**Judith Gecht, Ioannis Tsoukakis, Kim Kricheldorf, Frank Stegelmann, Martine Klausmann, Martin Griesshammer, Holger Schulz, Wiebke Hollburg, Joachim R. Göthert, Katja Sockel, Florian H. Heidel, Norbert Gattermann, Christoph Maintz, Haifa K. Al-Ali, Uwe Platzbecker, Richard Hansen, Mathias Hänel, Stefani Parmentier, Martin Bommer, Heike L. Pahl, Fabian Lang, Martin Kirschner, Susanne Isfort, Tim H. Brümmendorf, Konstanze Döhner and Steffen Koschmieder**

**Table S1.** Patient characteristics of the total cohort and by eGFR groups.

| Parameter<br>(*n*, % row, % column) | Category | total<br>(*n*, % in parameter) | eGFR1 | eGFR2 | eGFR3 | Comparison Between Pairs | *p* |
|---|---|---|---|---|---|---|---|
| Patients; *n*, % of total | - | 1420<br>100.0 | 304<br>21.4 | 799<br>56.3 | 317<br>22.3 | - | - |
| Diagnosis | - | - | - | - | - | -<br>ET - PV<br>eGFR1 - eGFR2<br>eGFR1 - eGFR3<br>eGFR2 - eGFR3<br>ET - MF<br>eGFR1 - eGFR2<br>eGFR1 - eGFR3<br>eGFR2 - eGFR3<br>PV - MF<br>eGFR1 - eGFR2<br>eGFR1 - eGFR3<br>eGFR2 - eGFR3 | **0.0188**<br>>0.9999<br>>0.9999<br>>0.9999<br>>0.9999<br>**0.0243**<br>0.8124<br>**0.0099**<br>0.0558<br>0.0795<br>>0.9999<br>0.0729<br>**0.0480** |
| | ET | 546<br>38.45 | 129<br>23.63<br>42.43 | 308<br>56.41<br>38.55 | 109<br>19.96<br>34.38 | | |
| | PV | 478<br>33.66 | 102<br>21.34<br>33.55 | 279<br>58.37<br>34.92 | 97<br>20.29<br>30.60 | | |
| | MF | 396<br>27.89 | 73<br>18.43<br>24.01 | 212<br>53.54<br>26.53 | 111<br>28.03<br>35.02 | | |
| Thromboembolism | | | | | | eGFR1 - eGFR2<br>eGFR1 - eGFR3<br>eGFR2 - eGFR3 | **0.0141**<br>>0.9999<br>**0.0453**<br>**0.0276** |
| | yes | 414<br>29.15 | 80<br>19.32<br>26.32 | 221<br>53.38<br>27.66 | 113<br>27.29<br>35.65 | | |
| | no | 1006<br>70.85 | 224<br>22.27<br>73.68 | 578<br>57.46<br>72.34 | 204<br>20.28<br>64.35 | | |
| Bleeding | | | | | | eGFR1 - eGFR2<br>eGFR1 - eGFR3<br>eGFR2 - eGFR3 | 0.6449<br>>0.9999<br>>0.9999<br>>0.9999 |
| | yes | 55<br>3.87 | 14<br>25.45<br>4.61 | 31<br>56.36<br>3.88 | 10<br>18.18<br>3.15 | | |
| | no | 1365<br>96.13 | 290<br>21.25<br>95.39 | 768<br>56.26<br>96.12 | 307<br>22.49<br>96.85 | | |
| Sex | | | | | | eGFR1 - eGFR2<br>eGFR1 - eGFR3<br>eGFR2 - eGFR3 | 0.0643<br>>0.9999<br>0.0741<br>0.2190 |
| | f | 726<br>51.13 | 144<br>19.83<br>47.37 | 403<br>55.51<br>50.44 | 179<br>24.66<br>56.47 | | |
| | m | 694<br>48.87 | 160<br>23.05<br>52.63 | 396<br>57.06<br>49.56 | 138<br>19.88<br>43.53 | | |
| Age at diagnosis, years | | | | | | eGFR1 - eGFR2<br>eGFR1 - eGFR3<br>eGFR2 - eGFR3 | **<0.0001**<br>**<0.0001**<br>**<0.0001**<br>**<0.0001** |
| | >60 | 606<br>42.80 | 80<br>13.20<br>26.40 | 318<br>52.48<br>39.80 | 208<br>34.32<br>66.24 | | |
| | ≤60 | 810<br>57.20 | 223<br>27.53<br>73.60 | 481<br>59.38<br>60.20 | 106<br>13.09<br>33.76 | | |
| Age at creatinine test, years | | | | | | eGFR1 - eGFR2<br>eGFR1 - eGFR3<br>eGFR2 - eGFR3 | **<0.0001**<br>**<0.0001**<br>**<0.0001**<br>**<0.0001** |
| | >60 | 780<br>54.93 | 103<br>13.21<br>33.88 | 416<br>53.33<br>52.07 | 261<br>33.46<br>82.33 | | |

| Parameter<br>(*n*, % row, % column) | Category | total<br>(*n*, % in parameter) | eGFR1 | eGFR2 | eGFR3 | Comparison Between Pairs | *p* |
|---|---|---|---|---|---|---|---|
| | ≤60 | 640<br>45.07 | 201<br>31.41<br>66.12 | 383<br>59.84<br>47.93 | 56<br>8.75<br>17.67 | | |
| Age at first MPN treatment, years | | | | | | | **<0.0001** |
| | >60 | 465<br>49.95 | 56<br>12.04<br>29.95 | 243<br>52.26<br>47.74 | 166<br>35.70<br>70.64 | eGFR1 - eGFR2<br>eGFR1 - eGFR3<br>eGFR2 - eGFR3 | **<0.0001**<br>**<0.0001**<br>**<0.0001** |
| | ≤60 | 466<br>50.05 | 131<br>28.11<br>70.05 | 266<br>57.08<br>52.26 | 69<br>14.81<br>29.36 | | |
| JAK2V617F mutation | | | | | | | 0.1014 |
| | yes | 964<br>73.31 | 208<br>21.58<br>71.48 | 533<br>55.29<br>72.12 | 223<br>23.13<br>78.25 | eGFR1 - eGFR2<br>eGFR1 - eGFR3<br>eGFR2 - eGFR3 | >0.9999<br>0.2046<br>0.1428 |
| | no | 351<br>26.69 | 83<br>23.65<br>28.52 | 206<br>58.69<br>27.88 | 62<br>17.66<br>21.75 | | |
| CALR mutation | | | | | | | **0.0192** |
| | yes | 139<br>27.31 | 39<br>28.06<br>32.23 | 82<br>58.99<br>29.18 | 18<br>12.95<br>16.82 | eGFR1 - eGFR2<br>eGFR1 - eGFR3<br>eGFR2 - eGFR3 | >0.9999<br>**0.0270**<br>**0.0405** |
| | no | 370<br>72.69 | 82<br>22.16<br>67.77 | 199<br>53.78<br>70.82 | 89<br>24.05<br>83.18 | | |
| Diabetes mellitus | | | | | | | **<0.0001** |
| | yes | 97<br>7.75 | 21<br>21.65<br>7.87 | 38<br>39.18<br>5.36 | 38<br>39.18<br>13.82 | eGFR1 - eGFR2<br>eGFR1 - eGFR3<br>eGFR2 - eGFR3 | 0.5223<br>0.0834<br>**<0.0001** |
| | No | 1154<br>92.25 | 246<br>21.32<br>92.13 | 671<br>58.15<br>94.64 | 237<br>20.54<br>86.18 | | |
| Arterial hypertension | | | | | | | **<0.0001** |
| | yes | 468<br>48.95 | 81<br>17.31<br>36.99 | 235<br>50.21<br>45.19 | 152<br>32.48<br>70.05 | eGFR1 - eGFR2<br>eGFR1 - eGFR3<br>eGFR2 - eGFR3 | 0.1260<br>**<0.0001**<br>**<0.0001** |
| | no | 488<br>51.05 | 138<br>28.28<br>63.01 | 285<br>58.40<br>54.81 | 65<br>13.32<br>29.95 | | |
| Hyperlipidemia | | | | | | | **0.0101** |
| | yes | 21<br>11.60 | 3<br>14.29<br>5.77 | 10<br>47.62<br>10.00 | 8<br>38.10<br>27.59 | eGFR1 - eGFR2<br>eGFR1 - eGFR3<br>eGFR2 - eGFR3 | >0.9999<br>**0.0420**<br>0.0873 |
| | no | 160<br>88.40 | 49<br>30.63<br>94.23 | 90<br>56.25<br>90.00 | 21<br>13.13<br>72.41 | | |
| ECOG-score [*,†] | | | | | | | **0.0184** |
| | ECOG 0 | 683<br>69.3 | 151<br>22.11<br>71.23 | 395<br>57.83<br>71.95 | 137<br>20.06<br>61.16 | eGFR1 - eGFR2<br>eGFR1 - eGFR3<br>eGFR2 - eGFR3 | >0.9999<br>0.0780<br>**0.0294** |
| | ECOG 1 | 270<br>27.4 | 52<br>19.26<br>24.53 | 141<br>52.22<br>25.68 | 77<br>28.52<br>34.38 | | |
| | ECOG 2 | 25<br>2.5 | 6<br>24.00<br>2.83 | 11<br>44.00<br>2.00 | 8<br>32.00<br>3.57 | | |
| | ECOG 3 | 7<br>0.7 | 3<br>42.86<br>1.42 | 2<br>28.57<br>0.36 | 2<br>28.57<br>0.89 | | |
| Leukocytes, G/L | | | | | | | **0.0013** |
| | >8.4 | 700<br>49.72 | 142<br>20.29<br>46.71 | 373<br>53.29<br>47.28 | 185<br>26.43<br>58.73 | eGFR1 - eGFR2<br>eGFR1 - eGFR3<br>eGFR2 - eGFR3 | >0.9999<br>**0.0087**<br>**0.0021** |
| | ≤8.4 | 708<br>50.28 | 162<br>22.88<br>53.29 | 416<br>58.76<br>52.72 | 130<br>18.36<br>41.27 | | |
| Platelets, G/L | | | | | | | **0.0283** |
| | >492 | 697<br>49.96 | 153<br>21.95<br>50.50 | 407<br>58.39<br>52.38 | 137<br>19.66<br>43.49 | eGFR1 - eGFR2<br>eGFR1 - eGFR3<br>eGFR2 - eGFR3 | >0.9999<br>0.2715<br>**0.0273** |

| Parameter<br>(*n*, % row, % column) | Category | total<br>(*n*, % in parameter) | eGFR1 | eGFR2 | eGFR3 | Comparison Between Pairs | *p* |
|---|---|---|---|---|---|---|---|
| | ≤492 | 698<br>50.04 | 150<br>21.49<br>49.50 | 370<br>53.01<br>47.62 | 178<br>25.50<br>56.51 | | |
| Uric acid, mg/dL | | | | | | | **<0.0001** |
| | >5.7 | 487<br>48.12 | 76<br>15.61<br>34.23 | 257<br>52.77<br>45.25 | 154<br>31.62<br>69.37 | eGFR1 - eGFR2<br>eGFR1 - eGFR3<br>eGFR2 - eGFR3 | **0.0150**<br>**<0.0001**<br>**<0.0001** |
| | ≤5.7 | 525<br>51.88 | 146<br>27.81<br>65.77 | 311<br>59.24<br>54.75 | 68<br>12.95<br>30.63 | | |
| Absolute neutrophil count, G/L | | | | | | | **0.0026** |
| | >5.48 | 625<br>49.84 | 125<br>20.00<br>45.96 | 339<br>54.24<br>47.81 | 161<br>25.76<br>58.97 | eGFR1 - eGFR2<br>eGFR1 - eGFR3<br>eGFR2 - eGFR3 | >0.9999<br>**0.0081**<br>**0.0051** |
| | ≤5.48 | 629<br>50.16 | 147<br>23.37<br>54.04 | 370<br>58.82<br>52.19 | 112<br>17.81<br>41.03 | | |
| Absolute monocyte count, G/L | | | | | | | **0.0014** |
| | >0.55 | 650<br>49.54 | 135<br>20.77<br>47.37 | 342<br>52.62<br>46.66 | 173<br>26.62<br>58.84 | eGFR1 - eGFR2<br>eGFR1 - eGFR3<br>eGFR2 - eGFR3 | >0.9999<br>**0.0180**<br>**0.0012** |
| | ≤0.55 | 662<br>50.46 | 150<br>22.66<br>52.63 | 391<br>59.06<br>53.34 | 121<br>18.28<br>41.16 | | |
| LDH, U/L | | | | | | | **0.0001** |
| | >267.5 | 678<br>50.00 | 128<br>18.88<br>43.84 | 369<br>54.42<br>48.30 | 181<br>26.70<br>60.33 | eGFR1 - eGFR2<br>eGFR1 - eGFR3<br>eGFR2 - eGFR3 | 0.6444<br>**0.0003**<br>**0.0015** |
| | ≤267.5 | 678<br>50.00 | 164<br>24.19<br>56.16 | 395<br>58.26<br>51.70 | 119<br>17.55<br>39.67 | | |
| CRP, mg/L | | | | | | | **0.0116** |
| | >1.4 | 172<br>48.31 | 35<br>20.35<br>44.30 | 85<br>49.42<br>43.81 | 52<br>30.23<br>62.65 | eGFR1 - eGFR2<br>eGFR1 - eGFR3<br>eGFR2 - eGFR3 | >0.9999<br>0.0813<br>**0.0171** |
| | ≤1.4 | 184<br>51.69 | 44<br>23.91<br>55.70 | 109<br>59.24<br>56.19 | 31<br>16.85<br>37.35 | | |

* ECOG 4 and 5 0% each. † Significance testing for ECOG 0 vs 1. Bolded values indicate significant *P* values.

**Table S2.** Patient characteristics by eGFR groups in ET patients

| Parameter<br>(*n*, % row, % column) | Category | total<br>(*n*, % in parameter) | eGFR1 | eGFR2 | eGFR3 | Comparison between pairs | *p* |
|---|---|---|---|---|---|---|---|
| Patients; *n*, % of total | | 546<br>100.0 | 129<br>23.63 | 308<br>56.41 | 109<br>19.96 | | |
| Thromboembolism | | | | | | | 0.3726 |
| | yes | 142<br>26.01 | 33<br>23.24<br>25.58 | 75<br>52.82<br>24.35 | 34<br>23.94<br>31.19 | eGFR1 - eGFR2<br>eGFR1 - eGFR3<br>eGFR2 - eGFR3 | >0.9999<br>>0.9999<br>0.4959 |
| | no | 404<br>73.99 | 96<br>23.76<br>74.42 | 233<br>57.67<br>75.65 | 75<br>18.56<br>68.81 | | |
| Bleeding | | | | | | | 0.2070 |
| | yes | 17<br>3.11 | 7<br>41.18<br>5.43 | 8<br>47.06<br>2.60 | 2<br>11.76<br>1.83 | eGFR1 - eGFR2<br>eGFR1 - eGFR3<br>eGFR2 - eGFR3 | 0.4629<br>0.5562<br>>0.9999 |
| | no | 529<br>96.89 | 122<br>23.06<br>94.57 | 300<br>56.71<br>97.40 | 107<br>20.23<br>98.17 | | |
| Sex | | | | | | | **0.0321** |
| | f | 319<br>58.42 | 63<br>19.75<br>48.84 | 186<br>58.31<br>60.39 | 70<br>21.94<br>64.22 | eGFR1 - eGFR2<br>eGFR1 - eGFR3<br>eGFR2 - eGFR3 | 0.1017<br>0.0564<br>>0.9999 |
| | m | 227<br>41.58 | 66<br>29.07<br>51.16 | 122<br>53.74<br>39.61 | 39<br>17.18<br>35.78 | | |
| Age at diagnosis, years | | | | | | | **<0.0001** |

| Parameter<br>(*n*, % row, % column) | Category | total<br>(*n*, % in parameter) | eGFR1 | eGFR2 | eGFR3 | Comparison between pairs | *p* |
|---|---|---|---|---|---|---|---|
| | >60 | 195<br>35.78 | 24<br>12.31<br>18.75 | 108<br>55.38<br>35.06 | 63<br>32.31<br>57.80 | eGFR1 - eGFR2<br>eGFR1 - eGFR3<br>eGFR2 - eGFR3 | **0.0024**<br>**<0.0001**<br>**<0.0001** |
| | ≤60 | 350<br>64.22 | 104<br>29.71<br>81.25 | 200<br>57.14<br>64.94 | 46<br>13.14<br>42.20 | | |
| Age at creatinine test, years | | | | | | | **<0.0001** |
| | >60 | 263<br>48.17 | 34<br>12.93<br>26.36 | 145<br>55.13<br>47.08 | 84<br>31.94<br>77.06 | eGFR1 - eGFR2<br>eGFR1 - eGFR3<br>eGFR2 - eGFR3 | **<0.0001**<br>**<0.0001**<br>**<0.0001** |
| | ≤60 | 283<br>51.83 | 95<br>33.57<br>73.64 | 163<br>57.60<br>52.92 | 25<br>8.83<br>22.94 | | |
| Age at first MPN treatment, years | | | | | | | **<0.0001** |
| | >60 | 163<br>45.53 | 17<br>10.43<br>20.73 | 95<br>58.28<br>47.98 | 51<br>31.29<br>65.38 | eGFR1 - eGFR2<br>eGFR1 - eGFR3<br>eGFR2 - eGFR3 | **<0.0001**<br>**<0.0001**<br>**0.0327** |
| | ≤60 | 195<br>54.47 | 65<br>33.33<br>79.27 | 103<br>52.82<br>52.02 | 27<br>13.85<br>34.62 | | |
| JAK2V617F mutation | | | | | | | **0.0032** |
| | yes | 317<br>63.15 | 66<br>20.82<br>54.55 | 176<br>55.52<br>62.19 | 75<br>23.66<br>76.53 | eGFR1 - eGFR2<br>eGFR1 - eGFR3<br>eGFR2 - eGFR3 | 0.5505<br>**0.0030**<br>**0.0291** |
| | no | 185<br>36.85 | 55<br>29.73<br>45.45 | 107<br>57.84<br>37.81 | 23<br>12.43<br>23.47 | | |
| CALR mutation | | | | | | | 0.1079 |
| | yes | 81<br>36.99 | 28<br>34.57<br>42.42 | 46<br>56.79<br>38.33 | 7<br>8.64<br>21.21 | eGFR1 - eGFR2<br>eGFR1 - eGFR3<br>eGFR2 - eGFR3 | >0.9999<br>0.1377<br>0.2922 |
| | no | 138<br>63.01 | 38<br>27.54<br>57.58 | 74<br>53.62<br>61.67 | 26<br>18.84<br>78.79 | | |
| Diabetes mellitus | | | | | | | **0.0001** |
| | yes | 31<br>6.40 | 8<br>25.81<br>7.02 | 8<br>25.81<br>2.92 | 15<br>48.39<br>15.63 | eGFR1 - eGFR2<br>eGFR1 - eGFR3<br>eGFR2 - eGFR3 | 0.2685<br>0.2226<br>**<0.0001** |
| | no | 453<br>93.60 | 106<br>23.40<br>92.98 | 266<br>58.72<br>97.08 | 81<br>17.88<br>84.38 | | |
| Arterial hypertension | | | | | | | **<0.0001** |
| | yes | 171<br>45.60 | 32<br>18.71<br>32.99 | 82<br>47.95<br>40.20 | 57<br>33.33<br>77.03 | eGFR1 - eGFR2<br>eGFR1 - eGFR3<br>eGFR2 - eGFR3 | 0.7611<br>**<0.0001**<br>**<0.0001** |
| | no | 204<br>54.40 | 65<br>31.86<br>67.01 | 122<br>59.80<br>59.80 | 17<br>8.33<br>22.97 | | |
| Hyperlipidemia | | | | | | | 0.3263 |
| | yes | 7<br>10.61 | 2<br>28.57<br>6.67 | 3<br>42.86<br>10.71 | 2<br>28.57<br>25.00 | eGFR1 - eGFR2<br>eGFR1 - eGFR3<br>eGFR2 - eGFR3 | >0.9999<br>0.5661<br>0.9144 |
| | no | 59<br>89.39 | 28<br>47.46<br>93.33 | 25<br>42.37<br>89.29 | 6<br>10.17<br>75.00 | | |
| ECOG-score [*,†] | | | | | | | 0.2586 |
| | ECOG 0 | 277<br>75.07 | 68<br>24.55<br>73.12 | 159<br>57.40<br>78.71 | 50<br>18.05<br>67.57 | eGFR1 - eGFR2<br>eGFR1 - eGFR3<br>eGFR2 - eGFR3 | >0.9999<br>>0.9999<br>0.4080 |
| | ECOG 1 | 82<br>22.22 | 21<br>25.61<br>22.58 | 40<br>48.78<br>19.80 | 21<br>25.61<br>28.38 | | |
| | ECOG 2 | 9<br>2.44 | 3<br>33.33<br>3.23 | 3<br>33.33<br>1.49 | 3<br>33.33<br>4.05 | | |
| | ECOG 3 | 1<br>0.27 | 1<br>100.00<br>1.08 | 0 | 0 | | |
| Leukocytes, G/L | | | | | | | 0.0685 |
| | >8.4 | 250 | 53 | 137 | 60 | eGFR1 - eGFR2 | >0.9999 |

| Parameter (*n*, % row, % column) | Category | total (*n*, % in parameter) | eGFR1 | eGFR2 | eGFR3 | Comparison between pairs | *p* |
|---|---|---|---|---|---|---|---|
| | | 46.13 | 21.20 | 54.80 | 24.00 | eGFR1 - eGFR3 | 0.0825 |
| | | | 41.09 | 44.92 | 55.56 | eGFR2 - eGFR3 | 0.2178 |
| | ≤8.4 | 292 | 76 | 168 | 48 | | |
| | | 53.87 | 26.03 | 57.53 | 16.44 | | |
| | | | 58.91 | 55.08 | 44.44 | | |
| Platelets, G/L | | | | | | | 0.0547 |
| | >492 | 409 | 103 | 233 | 73 | | |
| | | 76.02 | 25.18 | 56.97 | 17.85 | | |
| | | | 80.47 | 77.15 | 67.59 | eGFR1 - eGFR2 | >0.9999 |
| | ≤492 | 129 | 25 | 69 | 35 | eGFR1 - eGFR3 | 0.0762 |
| | | 23.98 | 19.38 | 53.49 | 27.13 | eGFR2 - eGFR3 | 0.1623 |
| | | | 19.53 | 22.85 | 32.41 | | |
| Uric acid, mg/dL | | | | | | | **<0.0001** |
| | >5.7 | 144 | 18 | 79 | 47 | | |
| | | 37.02 | 12.50 | 54.86 | 32.64 | | |
| | | | 19.78 | 35.43 | 62.67 | eGFR1 - eGFR2 | **0.0210** |
| | ≤5.7 | 245 | 73 | 144 | 28 | eGFR1 - eGFR3 | **<0.0001** |
| | | 62.98 | 29.80 | 58.78 | 11.43 | eGFR2 - eGFR3 | **<0.0001** |
| | | | 80.22 | 64.57 | 37.33 | | |
| Absolute neutrophil count, G/L | | | | | | | **0.0304** |
| | >5.48 | 226 | 43 | 129 | 54 | | |
| | | 45.75 | 19.03 | 57.08 | 23.89 | | |
| | | | 37.07 | 46.07 | 55.10 | eGFR1 - eGFR2 | 0.3564 |
| | ≤5.48 | 268 | 73 | 151 | 44 | eGFR1 - eGFR3 | **0.0273** |
| | | 54.25 | 27.24 | 56.34 | 16.42 | eGFR2 - eGFR3 | 0.3861 |
| | | | 62.93 | 53.93 | 44.90 | | |
| Absolute monocyte count, G/L | | | | | | | **0.0494** |
| | >0.55 | 263 | 52 | 149 | 62 | | |
| | | 51.27 | 19.77 | 56.65 | 23.57 | | |
| | | | 43.70 | 51.20 | 60.19 | eGFR1 - eGFR2 | 0.5751 |
| | ≤0.55 | 250 | 67 | 142 | 41 | eGFR1 - eGFR3 | **0.0474** |
| | | 48.73 | 26.80 | 56.80 | 16.40 | eGFR2 - eGFR3 | 0.4056 |
| | | | 56.30 | 48.80 | 39.81 | | |
| LDH, U/L | | | | | | | 0.2112 |
| | >267.5 | 184 | 38 | 102 | 44 | | |
| | | 34.72 | 20.65 | 55.43 | 23.91 | | |
| | | | 30.16 | 34.34 | 41.12 | eGFR1 - eGFR2 | >0.9999 |
| | ≤267.5 | 346 | 88 | 195 | 63 | eGFR1 - eGFR3 | 0.2958 |
| | | 65.28 | 25.43 | 56.36 | 18.21 | eGFR2 - eGFR3 | 0.7221 |
| | | | 69.84 | 65.66 | 58.88 | | |
| CRP, mg/L | | | | | | | 0.3321 |
| | >1.4 | 51 | 13 | 25 | 13 | | |
| | | 40.48 | 25.49 | 49.02 | 25.49 | | |
| | | | 33.33 | 40.32 | 52.00 | eGFR1 - eGFR2 | >0.9999 |
| | ≤1.4 | 75 | 26 | 37 | 12 | eGFR1 - eGFR3 | 0.5790 |
| | | 59.52 | 34.67 | 49.33 | 16.00 | eGFR2 - eGFR3 | >0.9999 |
| | | | 66.67 | 59.68 | 48.00 | | |

* ECOG 4 and 5 0% each. † Significance testing for ECOG 0 vs 1. Bolded values indicate significant *P* values.

**Table S3.** Patient characteristics by eGFR groups in PV patients

| Parameter (*n*, % row, % column) | Category | total (*n*, % in parameter) | eGFR1 | eGFR2 | eGFR3 | Comparison between pairs | *p* |
|---|---|---|---|---|---|---|---|
| Patients; *n*, % of total | | 478 | 102 | 279 | 97 | | |
| | | 100.0 | 21.34 | 58.37 | 20.29 | | |
| Thromboembolism | | | | | | | **0.0243** |
| | yes | 161 | 31 | 86 | 44 | | |
| | | 33.68 | 19.25 | 53.42 | 27.33 | | |
| | | | 30.39 | 30.82 | 45.36 | eGFR1 - eGFR2 | >0.9999 |
| | no | 317 | 71 | 193 | 53 | eGFR1 - eGFR3 | 0.1206 |
| | | 66.32 | 22.40 | 60.88 | 16.72 | eGFR2 - eGFR3 | **0.0387** |
| | | | 69.61 | 69.18 | 54.64 | | |
| Bleeding | | | | | | | 0.5696 |
| | yes | 20 | 5 | 13 | 2 | eGFR1 - eGFR2 | >0.9999 |
| | | 4.18 | 25.00 | 65.00 | 10.00 | eGFR1 - eGFR3 | >0.9999 |
| | | | 4.90 | 4.66 | 2.06 | eGFR2 - eGFR3 | >0.9999 |
| | no | 458 | 97 | 266 | 95 | | |
| | | 95.82 | 21.18 | 58.08 | 20.74 | | |

| Parameter (*n*, % row, % column) | Category | total (*n*, % in parameter) | eGFR1 | eGFR2 | eGFR3 | Comparison between pairs | *p* |
|---|---|---|---|---|---|---|---|
| | | | 95.10 | 95.34 | 97.94 | | |
| Sex | | | | | | | **0.0378** |
| | f | 240<br>50.21 | 44<br>18.33<br>43.14 | 137<br>57.08<br>49.10 | 59<br>24.58<br>60.82 | eGFR1 - eGFR2<br>eGFR1 - eGFR3<br>eGFR2 - eGFR3 | >0.9999<br>**0.0474**<br>0.1764 |
| | m | 238<br>49.79 | 58<br>24.37<br>56.86 | 142<br>59.66<br>50.90 | 38<br>15.97<br>39.18 | | |
| Age at diagnosis, years | | | | | | | **<0.0001** |
| | >60 | 224<br>46.96 | 30<br>13.39<br>29.41 | 125<br>55.80<br>44.80 | 69<br>30.80<br>71.88 | eGFR1 - eGFR2<br>eGFR1 - eGFR3<br>eGFR2 - eGFR3 | **0.0207**<br>**<0.0001**<br>**<0.0001** |
| | ≤60 | 253<br>53.04 | 72<br>28.46<br>70.59 | 154<br>60.87<br>55.20 | 27<br>10.67<br>28.13 | | |
| Age at creatinine test, years | | | | | | | **<0.0001** |
| | >60 | 293<br>61.30 | 41<br>13.99<br>40.20 | 165<br>56.31<br>59.14 | 87<br>29.69<br>89.69 | eGFR1 - eGFR2<br>eGFR1 - eGFR3<br>eGFR2 - eGFR3 | **0.0033**<br>**<0.0001**<br>**<0.0001** |
| | ≤60 | 185<br>38.70 | 61<br>32.97<br>59.80 | 114<br>61.62<br>40.86 | 10<br>5.41<br>10.31 | | |
| Age at first MPN treatment, years | | | | | | | **<0.0001** |
| | >60 | 171<br>55.88 | 20<br>11.70<br>33.90 | 93<br>54.39<br>53.45 | 58<br>33.92<br>79.45 | eGFR1 - eGFR2<br>eGFR1 - eGFR3<br>eGFR2 - eGFR3 | **0.0315**<br>**<0.0001**<br>**0.0003** |
| | ≤60 | 135<br>44.12 | 39<br>28.89<br>66.10 | 81<br>60.00<br>46.55 | 15<br>11.11<br>20.55 | | |
| JAK2V617F mutation | | | | | | | 0.2881 |
| | yes | 419<br>92.29 | 96<br>22.91<br>96.00 | 242<br>57.76<br>91.32 | 81<br>19.33<br>91.01 | eGFR1 - eGFR2<br>eGFR1 - eGFR3<br>eGFR2 - eGFR3 | 0.5328<br>0.6978<br>>0.9999 |
| | no | 35<br>7.71 | 4<br>11.43<br>4.00 | 23<br>65.71<br>8.68 | 8<br>22.86<br>8.99 | | |
| CALR mutation | | | | | | | 0.3973 |
| | yes | 2<br>1.63 | 1<br>50.00<br>4.55 | 1<br>50.00<br>1.47 | 0<br>0.00<br>0.00 | eGFR1 - eGFR2<br>eGFR1 - eGFR3<br>eGFR2 - eGFR3 | >0.9999<br>>0.9999<br>>0.9999 |
| | no | 121<br>98.37 | 21<br>17.36<br>95.45 | 67<br>55.37<br>98.53 | 33<br>27.27<br>100.00 | | |
| Diabetes mellitus | | | | | | | 0.4161 |
| | yes | 41<br>9.49 | 12<br>29.27<br>12.50 | 20<br>48.78<br>8.00 | 9<br>21.95<br>10.47 | eGFR1 - eGFR2<br>eGFR1 - eGFR3<br>eGFR2 - eGFR3 | 0.6459<br>>0.9999<br>>0.9999 |
| | no | 391<br>90.51 | 84<br>21.48<br>87.50 | 230<br>58.82<br>92.00 | 77<br>19.69<br>89.53 | | |
| Arterial hypertension | | | | | | | 0.0560 |
| | yes | 189<br>62.79 | 35<br>18.52<br>53.03 | 105<br>55.56<br>62.50 | 49<br>25.93<br>73.13 | eGFR1 - eGFR2<br>eGFR1 - eGFR3<br>eGFR2 - eGFR3 | 0.5604<br>0.0594<br>0.3936 |
| | no | 112<br>37.21 | 31<br>27.68<br>46.97 | 63<br>56.25<br>37.50 | 18<br>16.07<br>26.87 | | |
| Hyperlipidemia | | | | | | | 0.5809 |
| | yes | 8<br>17.02 | 1<br>12.50<br>9.09 | 6<br>75.00<br>18.18 | 1<br>12.50<br>33.33 | eGFR1 - eGFR2<br>eGFR1 - eGFR3<br>eGFR2 - eGFR3 | >0.9999<br>>0.9999<br>>0.9999 |
| | no | 39<br>82.98 | 10<br>25.64<br>90.91 | 27<br>69.23<br>81.82 | 2<br>5.13<br>66.67 | | |
| ECOG-score *,† | | | | | | | 0.3696 |
| | ECOG 0 | 236<br>70.87 | 50<br>21.19<br>76.92 | 133<br>56.36<br>67.86 | 53<br>22.46<br>73.61 | eGFR1 - eGFR2<br>eGFR1 - eGFR3<br>eGFR2 - eGFR3 | 0.6075<br>>0.9999<br>>0.9999 |
| | ECOG 1 | 92<br>27.63 | 14<br>15.22 | 59<br>64.13 | 19<br>20.65 | | |

| Parameter<br>(*n*, % row, % column) | Category | total<br>(*n*, % in parameter) | eGFR1 | eGFR2 | eGFR3 | Comparison between pairs | *p* |
|---|---|---|---|---|---|---|---|
| | | | 21.54 | 30.10 | 26.39 | | |
| | ECOG 2 | 4<br>1.20 | 1<br>25.00<br>1.54 | 3<br>75.00<br>1.53 | 0 | | |
| | ECOG 3 | 1<br>0.30 | 0 | 1<br>100.00<br>0.51 | 0 | | |
| Leukocytes, G/L | | | | | | | 0.4807 |
| | >8.4 | 269<br>57.11 | 54<br>20.07<br>52.94 | 156<br>57.99<br>57.14 | 59<br>21.93<br>61.46 | eGFR1 - eGFR2<br>eGFR1 - eGFR3<br>eGFR2 - eGFR3 | >0.9999<br>0.7563<br>>0.9999 |
| | ≤8.4 | 202<br>42.89 | 48<br>23.76<br>47.06 | 117<br>57.92<br>42.86 | 37<br>18.32<br>38.54 | | |
| Platelets, G/L | | | | | | | 0.5275 |
| | >492 | 173<br>37.12 | 33<br>19.08<br>32.35 | 103<br>59.54<br>38.58 | 37<br>21.39<br>38.14 | eGFR1 - eGFR2<br>eGFR1 - eGFR3<br>eGFR2 - eGFR3 | 0.8406<br>>0.9999<br>>0.9999 |
| | ≤492 | 293<br>62.88 | 69<br>23.55<br>67.65 | 164<br>55.97<br>61.42 | 60<br>20.48<br>61.86 | | |
| Uric acid, mg/dL | | | | | | | **0.0014** |
| | >5.7 | 186<br>51.24 | 34<br>18.28<br>40.96 | 101<br>54.30<br>49.03 | 51<br>27.42<br>68.92 | eGFR1 - eGFR2<br>eGFR1 - eGFR3<br>eGFR2 - eGFR3 | 0.7251<br>**0.0021**<br>**0.0123** |
| | ≤5.7 | 177<br>48.76 | 49<br>27.68<br>59.04 | 105<br>59.32<br>50.97 | 23<br>12.99<br>31.08 | | |
| Absolute neutrophil count, G/L | | | | | | | 0.2392 |
| | >5.48 | 245<br>60.05 | 52<br>21.22<br>57.14 | 139<br>56.73<br>58.40 | 54<br>22.04<br>68.35 | eGFR1 - eGFR2<br>eGFR1 - eGFR3<br>eGFR2 - eGFR3 | >0.9999<br>0.4632<br>0.4296 |
| | ≤5.48 | 163<br>39.95 | 39<br>23.93<br>42.86 | 99<br>60.74<br>41.60 | 25<br>15.34<br>31.65 | | |
| Absolute monocyte count, G/L | | | | | | | **0.0244** |
| | >0.55 | 218<br>50.46 | 54<br>24.77<br>55.67 | 111<br>50.92<br>44.94 | 53<br>24.31<br>60.23 | eGFR1 - eGFR2<br>eGFR1 - eGFR3<br>eGFR2 - eGFR3 | 0.2787<br>>0.9999<br>0.0540 |
| | ≤0.55 | 214<br>49.54 | 43<br>20.09<br>44.33 | 136<br>63.55<br>55.06 | 35<br>16.36<br>39.77 | | |
| LDH, U/L | | | | | | | **0.0466** |
| | >267.5 | 205<br>45.66 | 43<br>20.98<br>43.88 | 111<br>54.15<br>42.37 | 51<br>24.88<br>57.30 | eGFR1 - eGFR2<br>eGFR1 - eGFR3<br>eGFR2 - eGFR3 | >0.9999<br>0.2382<br>0.0573 |
| | ≤267.5 | 244<br>54.34 | 55<br>22.54<br>56.12 | 151<br>61.89<br>57.63 | 38<br>15.57<br>42.70 | | |
| CRP, mg/L | | | | | | | 0.3641 |
| | >1.4 | 55<br>45.45 | 10<br>18.18<br>43.48 | 31<br>56.36<br>41.89 | 14<br>25.45<br>58.33 | eGFR1 - eGFR2<br>eGFR1 - eGFR3<br>eGFR2 - eGFR3 | >0.9999<br>>0.9999<br>0.7149 |
| | ≤1.4 | 66<br>54.55 | 13<br>19.70<br>56.52 | 43<br>65.15<br>58.11 | 10<br>15.15<br>41.67 | | |

* ECOG 4 and 5 0% each. † Significance testing for ECOG 0 vs 1. Bolded values indicate significant *P* values.

**Table S4.** Patient characteristics by eGFR groups in MF patients

| Parameter<br>(*n*, % row, % column) | Category | total<br>(*n*, % in parameter) | eGFR1 | eGFR2 | eGFR3 | Comparison between pairs | *p* |
|---|---|---|---|---|---|---|---|
| Patients; *n*, % of total | | 396<br>100.0 | 73<br>18.43 | 212<br>53.54 | 111<br>28.03 | | |
| Thromboembolism | | | | | | | 0.3616 |
| | yes | 111<br>28.03 | 16<br>14.41<br>21.92 | 60<br>54.05<br>28.30 | 35<br>31.53<br>31.53 | eGFR1 - eGFR2<br>eGFR1 - eGFR3<br>eGFR2 - eGFR3 | >0.9999<br>0.5391<br>>0.9999 |

| Parameter<br>(*n*, % row, % column) | Category | total<br>(*n*, % in parameter) | eGFR1 | eGFR2 | eGFR3 | Comparison between pairs | *p* |
|---|---|---|---|---|---|---|---|
| | no | 285<br>71.97 | 57<br>20.00<br>78.08 | 152<br>53.33<br>71.70 | 76<br>26.67<br>68.47 | | |
| Bleeding | | | | | | | 0.6865 |
| | yes | 18<br>4.55 | 2<br>11.11<br>2.74 | 10<br>55.56<br>4.72 | 6<br>33.33<br>5.41 | eGFR1 - eGFR2<br>eGFR1 - eGFR3<br>eGFR2 - eGFR3 | >0.9999<br>>0.9999<br>>0.9999 |
| | no | 378<br>95.45 | 71<br>18.78<br>97.26 | 202<br>53.44<br>95.28 | 105<br>27.78<br>94.59 | | |
| Sex | | | | | | | 0.1191 |
| | f | 167<br>42.17 | 37<br>22.16<br>50.68 | 80<br>47.90<br>37.74 | 50<br>29.94<br>45.05 | eGFR1 - eGFR2<br>eGFR1 - eGFR3<br>eGFR2 - eGFR3 | 0.1653<br>>0.9999<br>0.6975 |
| | m | 229<br>57.83 | 36<br>15.72<br>49.32 | 132<br>57.64<br>62.26 | 61<br>26.64<br>54.95 | | |
| Age at diagnosis, years | | | | | | | **<0.0001** |
| | >60 | 187<br>47.46 | 26<br>13.90<br>35.62 | 85<br>45.45<br>40.09 | 76<br>40.64<br>69.72 | eGFR1 - eGFR2<br>eGFR1 - eGFR3<br>eGFR2 - eGFR3 | >0.9999<br>**<0.0001**<br>**<0.0001** |
| | ≤60 | 207<br>52.54 | 47<br>22.71<br>64.38 | 127<br>61.35<br>59.91 | 33<br>15.94<br>30.28 | | |
| Age at creatinine test, years | | | | | | | **<0.0001** |
| | >60 | 224<br>56.57 | 28<br>12.50<br>38.36 | 106<br>47.32<br>50.00 | 90<br>40.18<br>81.08 | eGFR1 - eGFR2<br>eGFR1 - eGFR3<br>eGFR2 - eGFR3 | 0.3084<br>**<0.0001**<br>**<0.0001** |
| | ≤60 | 172<br>43.43 | 45<br>26.16<br>61.64 | 106<br>61.63<br>50.00 | 21<br>12.21<br>18.92 | | |
| Age at first MPN treatment, years | | | | | | | **0.0002** |
| | >60 | 131<br>49.06 | 19<br>14.50<br>41.30 | 55<br>41.98<br>40.15 | 57<br>43.51<br>67.86 | eGFR1 - eGFR2<br>eGFR1 - eGFR3<br>eGFR2 - eGFR3 | >0.9999<br>**0.0015**<br>**<0.0001** |
| | ≤60 | 136<br>50.94 | 27<br>19.85<br>58.70 | 82<br>60.29<br>59.85 | 27<br>19.85<br>32.14 | | |
| JAK2V617F mutation | | | | | | | 0.3602 |
| | yes | 228<br>63.51 | 46<br>20.18<br>65.71 | 115<br>50.44<br>60.21 | 67<br>29.39<br>68.37 | eGFR1 - eGFR2<br>eGFR1 - eGFR3<br>eGFR2 - eGFR3 | >0.9999<br>>0.9999<br>0.5967 |
| | no | 131<br>36.49 | 24<br>18.32<br>34.29 | 76<br>58.02<br>39.79 | 31<br>23.66<br>31.63 | | |
| CALR mutation | | | | | | | 0.4311 |
| | yes | 56<br>33.53 | 10<br>17.86<br>30.30 | 35<br>62.50<br>37.63 | 11<br>19.64<br>26.83 | eGFR1 - eGFR2<br>eGFR1 - eGFR3<br>eGFR2 - eGFR3 | >0.9999<br>>0.9999<br>0.7308 |
| | no | 111<br>66.47 | 23<br>20.72<br>69.70 | 58<br>52.25<br>62.37 | 30<br>27.03<br>73.17 | | |
| Diabetes mellitus | | | | | | | **0.0031** |
| | yes | 25<br>7.46 | 1<br>4.00<br>1.75 | 10<br>40.00<br>5.41 | 14<br>56.00<br>15.05 | eGFR1 - eGFR2<br>eGFR1 - eGFR3<br>eGFR2 - eGFR3 | >0.9999<br>**0.0288**<br>**0.0342** |
| | no | 310<br>92.54 | 56<br>18.06<br>98.25 | 175<br>56.45<br>94.59 | 79<br>25.48<br>84.95 | | |
| Arterial hypertension | | | | | | | **<0.0001** |
| | yes | 108<br>38.57 | 14<br>12.96<br>25.00 | 48<br>44.44<br>32.43 | 46<br>42.59<br>60.53 | eGFR1 - eGFR2<br>eGFR1 - eGFR3<br>eGFR2 - eGFR3 | >0.9999<br>**<0.0001**<br>**<0.0001** |
| | no | 172<br>61.43 | 42<br>24.42<br>75.00 | 100<br>58.14<br>67.57 | 30<br>17.44<br>39.47 | | |
| Hyperlipidemia | | | | | | | **0.0041** |
| | yes | 6<br>8.82 | 0<br>0.00 | 1<br>16.67 | 5<br>83.33 | eGFR1 - eGFR2<br>eGFR1 - eGFR3 | >0.9999<br>0.3783 |

| **Parameter**<br>**(*n*, % row, % column)** | **Category** | **total**<br>**(*n*, % in parameter)** | **eGFR1** | **eGFR2** | **eGFR3** | **Comparison between pairs** | ***p*** |
|---|---|---|---|---|---|---|---|
| | | | 0.00 | 2.5 | 27.78 | eGFR2 - eGFR3 | **0.0291** |
| | no | 62<br>91.18 | 11<br>17.74<br>100.00 | 38<br>61.29<br>97.44 | 13<br>20.97<br>72.22 | | |
| ECOG-score *, † | | | | | | | **0.0042** |
| | ECOG 0 | 170<br>60.07 | 33<br>19.41<br>61.11 | 103<br>60.59<br>68.21 | 34<br>20.00<br>43.59 | eGFR1-eGFR2<br>eGFR1-eGFR3<br>eGFR2-eGFR3 | >0.9999<br>0.1902<br>**0.0045** |
| | ECOG 1 | 96<br>33.92 | 17<br>17.71<br>31.48 | 42<br>43.75<br>27.81 | 37<br>38.54<br>47.44 | | |
| | ECOG 2 | 12<br>4.24 | 2<br>16.67<br>3.70 | 5<br>41.67<br>3.31 | 5<br>41.67<br>6.41 | | |
| | ECOG 3 | 5<br>1.77 | 2<br>40.00<br>3.70 | 1<br>20.00<br>0.66 | 2<br>40.00<br>2.56 | | |
| Leukocytes, G/L | | | | | | | **0.0010** |
| | >8.4 | 181<br>45.82 | 35<br>19.34<br>47.95 | 80<br>44.20<br>37.91 | 66<br>36.46<br>59.46 | eGFR1 - eGFR2<br>eGFR1 - eGFR3<br>eGFR2 - eGFR3 | 0.4992<br>0.3987<br>**0.0009** |
| | ≤8.4 | 214<br>54.18 | 38<br>17.76<br>52.05 | 131<br>61.21<br>62.09 | 45<br>21.03<br>40.54 | | |
| Platelets, G/L | | | | | | | 0.0904 |
| | >492 | 115<br>29.41 | 17<br>14.78<br>23.29 | 71<br>61.74<br>34.13 | 27<br>23.48<br>24.55 | eGFR1 - eGFR2<br>eGFR1 - eGFR3<br>eGFR2 - eGFR3 | 0.3192<br>>0.9999<br>0.2904 |
| | ≤492 | 276<br>70.59 | 56<br>20.29<br>76.71 | 137<br>49.64<br>65.87 | 83<br>30.07<br>75.45 | | |
| Uric acid, mg/dL | | | | | | | **0.0028** |
| | >5.7 | 157<br>60.38 | 24<br>15.29<br>50.00 | 77<br>49.04<br>55.40 | 56<br>35.67<br>76.71 | eGFR1 - eGFR2<br>eGFR1 - eGFR3<br>eGFR2 - eGFR3 | >0.9999<br>**0.0096**<br>**0.0081** |
| | ≤5.7 | 103<br>39.62 | 24<br>23.30<br>50.00 | 62<br>60.19<br>44.60 | 17<br>16.50<br>23.29 | | |
| Absolute neutrophil count, G/L | | | | | | | **0.0134** |
| | >5.48 | 154<br>43.75 | 30<br>19.48<br>46.15 | 71<br>46.10<br>37.17 | 53<br>34.42<br>55.21 | eGFR1 - eGFR2<br>eGFR1 - eGFR3<br>eGFR2 - eGFR3 | 0.7200<br>0.7992<br>**0.0159** |
| | ≤5.48 | 198<br>56.25 | 35<br>17.68<br>53.85 | 120<br>60.61<br>62.83 | 43<br>21.72<br>44.79 | | |
| Absolute monocyte count, G/L | | | | | | | **0.0481** |
| | >0.55 | 169<br>46.05 | 29<br>17.16<br>42.03 | 82<br>48.52<br>42.05 | 58<br>34.32<br>56.31 | eGFR1 - eGFR2<br>eGFR1 - eGFR3<br>eGFR2 - eGFR3 | >0.9999<br>0.2604<br>0.0624 |
| | ≤0.55 | 198<br>53.95 | 40<br>20.20<br>57.97 | 113<br>57.07<br>57.95 | 45<br>22.73<br>43.69 | | |
| LDH, U/L | | | | | | | 0.1157 |
| | >267.5 | 289<br>76.66 | 47<br>16.26<br>69.12 | 156<br>53.98<br>76.10 | 86<br>29.76<br>82.69 | eGFR1 - eGFR2<br>eGFR1 - eGFR3<br>eGFR2 - eGFR3 | 0.7944<br>0.1269<br>0.5781 |
| | ≤267.5 | 88<br>23.34 | 21<br>23.86<br>30.88 | 49<br>55.68<br>23.90 | 18<br>20.45<br>17.31 | | |
| CRP, mg/L | | | | | | | 0.0545 |
| | >1.4 | 66<br>60.55 | 12<br>18.18<br>70.59 | 29<br>43.94<br>50.00 | 25<br>37.88<br>73.53 | eGFR1 - eGFR2<br>eGFR1 - eGFR3<br>eGFR2 - eGFR3 | 0.5130<br>>0.9999<br>0.0915 |
| | ≤1.4 | 43<br>39.45 | 5<br>11.63<br>29.41 | 29<br>67.44<br>50.00 | 9<br>20.93<br>26.47 | | |

* ECOG 4 and 5 0% each. † Significance testing for ECOG 0 vs 1. Bolded values indicate significant *P* values.

**Table S5.** Correlations between uric acid, leukocytes, LDH, and platelets

| **MPN subtype** | **Uric acid, mg/dL** | **Leukocytes, G/L** | | **LDH, U/L** | | **Platelets, G/L** | | **Significance** |
|---|---|---|---|---|---|---|---|---|
| **(*n*, % row, % column)** | **(*n*, % subgroup)** | **>8.4** | **≤8.4** | **>267.5** | **≤267.5** | **>492** | **≤492** | |
| All patients (*n* = 1012) | | | | | | | | |
| | >5.7 | 283 | 197 | 272 | 196 | 210 | 268 | |
| all eGFR groups | 487, 48.12 | 58.96 | 41.04 | 58.12 | 41.88 | 43.93 | 56.07 | EU//LDH/PLT |
| | | 55.49 | 40.12 | 55.28 | 40.33 | 39.55 | 58.26 | |
| | ≤5.7 | 227 | 294 | 220 | 290 | 321 | 192 | |
| | 525, 51.88 | 43.57 | 56.43 | 43.14 | 56.86 | 62.57 | 37.43 | |
| | | 44.51 | 59.88 | 44.72 | 59.67 | 60.45 | 41.74 | |
| | >5.7 | 186 | 141 | 170 | 148 | 143 | 182 | |
| eGFR1/eGFR2 | 333, 42.15 | 56.88 | 43.12 | 53.46 | 46.54 | 44.00 | 56.00 | LEU/LDH/PLT |
| | | 48.69 | 35.43 | 47.22 | 36.72 | 33.73 | 52.60 | |
| | ≤5.7 | 196 | 257 | 190 | 255 | 281 | 164 | |
| | 457, 57.85 | 43.27 | 56.73 | 42.70 | 57.30 | 63.15 | 36.85 | |
| | | 51.31 | 64.57 | 52.78 | 63.28 | 66.27 | 47.40 | |
| | >5.7 | 97 | 56 | 102 | 48 | 67 | 86 | |
| eGFR3 | 154, 69.37 | 63.40 | 36.60 | 68.00 | 32.00 | 43.79 | 56.21 | LEU/LDH/PLT |
| | | 75.78 | 60.22 | 77.27 | 57.83 | 62.62 | 75.44 | |
| | ≤5.7 | 31 | 37 | 30 | 35 | 40 | 28 | |
| | 68, 30.63 | 45.59 | 54.41 | 46.15 | 53.85 | 58.82 | 41.18 | |
| | | 24.22 | 39.78 | 22.73 | 42.17 | 37.38 | 24.56 | |
| ET (*n* = 389) | | | | | | | | |
| | >5.7 | 78 | 64 | 56 | 85 | 106 | 37 | |
| eGFR1-3 | 144, 37.02 | 54.93 | 45.07 | 39.72 | 60.28 | 74.13 | 25.87 | LEU/PLT |
| | | 43.33 | 31.07 | 40.88 | 34.98 | 34.19 | 50.00 | |
| | ≤5.7 | 102 | 142 | 81 | 158 | 204 | 37 | |
| | 245, 62.98 | 41.80 | 58.20 | 33.89 | 66.11 | 84.65 | 15.35 | |
| | | 56.67 | 68.93 | 59.12 | 65.02 | 65.81 | 50.00 | |
| | >5.7 | 52 | 43 | 34 | 60 | 77 | 19 | |
| eGFR1/eGFR2 | 97, 30.89 | 54.74 | 45.26 | 36.17 | 63.83 | 80.21 | 19.79 | LEU |
| | | 37.14 | 25.15 | 32.38 | 30.00 | 29.96 | 36.54 | |
| | ≤5.7 | 88 | 128 | 71 | 140 | 180 | 33 | |
| | 217, 69.11 | 40.74 | 59.26 | 33.65 | 66.35 | 84.51 | 15.49 | |
| | | 62.86 | 74.85 | 67.62 | 70.00 | 70.04 | 63.46 | |
| | >5.7 | 26 | 21 | 22 | 25 | 29 | 18 | |
| eGFR3 | 47, 62.67 | 55.32 | 44.68 | 46.81 | 53.19 | 61.70 | 38.30 | PLT |
| | | 65.00 | 60.00 | 68.75 | 58.14 | 54.72 | 81.82 | |
| | ≤5.7 | 14 | 14 | 10 | 18 | 24 | 4 | |
| | 28, 37.33 | 50.00 | 50.00 | 35.71 | 64.29 | 85.71 | 14.29 | |
| | | 35.00 | 40.00 | 31.25 | 41.86 | 45.28 | 18.18 | |
| PV (*n* = 363) | | | | | | | | |
| | >5.7 | 124 | 58 | 91 | 84 | 57 | 123 | |
| eGFR1-3 | 186, 51.24 | 68.13 | 31.87 | 52.00 | 48.00 | 31.67 | 68.33 | LEU/LDH/PLT |
| | | 60.49 | 38.41 | 57.59 | 44.68 | 42.54 | 56.68 | |
| | ≤5.7 | 81 | 93 | 67 | 104 | 77 | 94 | |
| | 177, 48.76 | 46.55 | 53.45 | 39.18 | 60.82 | 45.03 | 54.97 | |
| | | 39.51 | 61.59 | 42.41 | 55.32 | 57.46 | 43.32 | |
| | >5.7 | 90 | 42 | 58 | 69 | 34 | 95 | |
| eGFR1/eGFR2 | 135, 46.71 | 68.18 | 31.82 | 45.67 | 54.33 | 26.36 | 73.64 | LEU/PLT |
| | | 55.56 | 34.71 | 49.15 | 43.67 | 33.66 | 53.98 | |
| | ≤5.7 | 72 | 79 | 60 | 89 | 67 | 81 | |
| | 154, 53.29 | 47.68 | 52.32 | 40.27 | 59.73 | 45.27 | 54.73 | |
| | | 44.44 | 65.29 | 50.85 | 56.33 | 66.34 | 46.02 | |
| | >5.7 | 34 | 16 | 33 | 15 | 23 | 28 | |
| eGFR3 | 51, 68.92 | 68.00 | 32.00 | 68.75 | 31.25 | 45.10 | 54.90 | LEU/LDH |
| | | 79.07 | 53.33 | 82.50 | 50.00 | 69.70 | 68.29 | |
| | ≤5.7 | 9 | 14 | 7 | 15 | 10 | 13 | |
| | 23, 31.08 | 39.13 | 60.87 | 31.82 | 68.18 | 43.48 | 56.52 | |
| | | 20.93 | 46.67 | 17.50 | 50.00 | 30.30 | 31.71 | |
| MF (*n* = 260) | | | | | | | | |
| | >5.7 | 81 | 75 | 125 | 27 | 47 | 108 | |
| eGFR1-3 | 157, 60.38 | 51.92 | 48.08 | 82.24 | 17.76 | 30.32 | 69.68 | |
| | | 64.80 | 55.97 | 63.45 | 49.09 | 54.02 | 63.91 | |
| | ≤5.7 | 44 | 59 | 72 | 28 | 40 | 61 | |
| | 103, 39.62 | 42.72 | 57.28 | 72.00 | 28.00 | 39.60 | 60.40 | |
| | | 35.20 | 44.03 | 36.55 | 50.91 | 45.98 | 36.09 | |
| | >5.7 | 44 | 56 | 78 | 19 | 32 | 68 | |
| eGFR1/eGFR2 | 101, 54.01 | 44.00 | 56.00 | 80.41 | 19.59 | 32.00 | 68.00 | |
| | | 55.00 | 52.83 | 56.93 | 42.22 | 48.48 | 57.63 | |

| MPN subtype (*n*, % row, % column) | Uric acid, mg/dL (*n*, % subgroup) | Leukocytes, G/L | | LDH, U/L | | Platelets, G/L | | Significance |
|---|---|---|---|---|---|---|---|---|
| | | >8.4 | ≤8.4 | >267.5 | ≤267.5 | >492 | ≤492 | |
| eGFR3 | ≤5.7<br>86, 45.99 | 36<br>41.86<br>45.00 | 50<br>58.14<br>47.17 | 59<br>69.41<br>43.07 | 26<br>30.59<br>57.78 | 34<br>40.48<br>51.52 | 50<br>59.52<br>42.37 | |
| | >5.7<br>56, 76.71 | 37<br>66.07<br>82.22 | 19<br>33.93<br>67.86 | 47<br>85.45<br>78.33 | 8<br>14.55<br>80.00 | 15<br>27.27<br>71.43 | 40<br>72.73<br>78.43 | |
| | ≤5.7<br>17, 23.29 | 8<br>47.06<br>17.78 | 9<br>52.94<br>32.14 | 13<br>86.67<br>21.67 | 2<br>13.33<br>20.00 | 6<br>35.29<br>28.57 | 11<br>64.71<br>21.57 | |

LEU indicates leukocytes; and PLT, platelets. Bolded values indicate significant *P* values.

**Table S6.** Median MPN duration across eGFR groups.

| MPN duration, months (*n* = 1183) | eGFR1 | eGFR2 | eGFR3 | Total | Comparison between pairs | *p* |
|---|---|---|---|---|---|---|
| *n* | 244 | 660 | 279 | 1183 | | |
| Median | 31.5 | 43.5 | 57.0 | 42.0 | | |
| Q1 | 5.0 | 11.5 | 19.0 | 11.0 | | |
| Q3 | 77.5 | 104.0 | 132.0 | 105.0 | | |
| | | | | | | **0.0002** |
| | | | | | eGFR1 - eGFR2 | **0.0255** |
| | | | | | eGFR1 - eGFR3 | **<0.0003** |
| | | | | | eGFR2 - eGFR3 | 0.0786 |

Bolded values indicate significant *P* values.

**Table S7.** MPN duration of >42 months versus ≤42 months across eGFR groups.

| MPN duration, months (*n* = 1183) (*n*, % row, % column) | eGFR1 | eGFR2 | eGFR3 | Total | Comparison between pairs | *p* |
|---|---|---|---|---|---|---|
| >42 | 99<br>16.81<br>40.57 | 332<br>56.37<br>50.30 | 158<br>26.83<br>56.63 | 589<br>49.79 | | |
| ≤42 | 145<br>24.41<br>59.43 | 328<br>55.22<br>49.70 | 121<br>20.37<br>43.37 | 594<br>50.21 | | |
| Total | 244<br>20.63 | 660<br>55.79 | 279<br>23.58 | 1183<br>100.00 | | |
| | | | | | | **0.0011** |
| | | | | | eGFR1 - eGFR2 | **0.0321** |
| | | | | | eGFR1 - eGFR3 | **0.0009** |
| | | | | | eGFR2 - eGFR3 | 0.2586 |

Bolded values indicate significant *P* values.

**Table S8.** Median MPN duration across creatinine groups.

| MPN duration, months (*n* = 1183) | Creatinine level, mg/dL | | Total | *p* |
|---|---|---|---|---|
| | >0.9 | ≤0.9 | | |
| *n* | 574 | 609 | 1183 | |
| Median | 49.00 | 37.00 | 42.00 | |
| Q1 | 13.00 | 8.00 | 11.00 | |
| Q3 | 117.00 | 94.00 | 105.00 | |
| | | | | **0.0198** |

Bolded values indicate significant *P* values.

**Table S9.** MPN duration of >42 months versus ≤42 months across creatinine groups.

| MPN duration, months (*n* = 1183) | Creatinine level, mg/dL (*n*, % row, % column) | | Total | *p* |
|---|---|---|---|---|
| | >0.9 | ≤0.9 | | |
| **>42** | 304<br>52.96<br>51.61 | 285<br>46.80<br>48.39 | 589<br>49.79 | |
| **≤42** | 270<br>47.04<br>45.45 | 324<br>53.20<br>54.55 | 594<br>50.21 | |
| **Total** | 574 | 609 | 1183 | |

| | | | |
|---|---|---|---|
| 48.52 | 51.48 | 100.00 | |
| | | | **0.0363** |

Bolded values indicate significant *P* values.

**Table S10.** Risk factors for MPN duration longer than 42 months.

| Risk factors for MPN duration >42 months | Odds ratio | 95% CI | *p* |
|---|---|---|---|
| Univariate regression | | | |
| Diagnosis (*n* = 1183) | | | |
| ET vs PV (*n* = 828) | 0.818 | 0.621 - 1.076 | 0.1502 |
| ET vs MF (*n* = 787) | **1.709** | **1.285 - 2.273** | **0.0002** |
| MF vs PV (*n* = 751) | **0.478** | **0.357 - 0.640** | **<0.0001** |
| Sex (female vs male; *n* = 1183) | 1.239 | 0.986 - 1.556 | 0.0664 |
| JAK2V617F mutation (yes vs no; *n* = 1100) | 0.891 | 0.683 - 1.163 | 0.3963 |
| Diabetes mellitus (yes vs no; *n* = 1019) | **0.541** | **0.325 - 0.900** | **0.0181** |
| Arterial hypertension (yes vs no; *n* = 829) | **1.531** | **1.163 - 2.017** | **0.0024** |
| Hyperlipidemia (yes vs no; *n* = 162) | 1.653 | 0.616 - 4.434 | 0.3179 |
| Leukocytes (>8.4 vs ≤8.4 G/L; *n* = 1181) | **0.769** | **0.611 - 0.966** | **0.0243** |
| Platelets (>492 vs ≤492 G/L; *n* = 1169) | 0.844 | 0.671 - 1.063 | 0.1494 |
| Uric acid (>5.7 vs ≤5.7 mg/dL; *n* = 799) | 1.106 | 0.838 - 1.460 | 0.4770 |
| Absolute neutrophil count (>5.48 vs ≤5.48 G/L; *n* = 1052) | 0.862 | 0.676 - 1.098 | 0.2293 |
| Absolute monocyte count (>0.55 vs ≤0.55 G/L; *n* = 1106) | 0.954 | 0.753 - 1.208 | 0.6954 |
| LDH (>267.5 vs ≤267.5 U/L; *n* = 1141) | 1.017 | 0.807 - 1.283 | 0.8836 |
| CRP (>1.4 vs ≤1.4 mg/L; *n* = 332) | 1.142 | 0.739 - 1.763 | 0.5500 |
| MPN therapy (yes vs WW; *n* = 1087) | **3.546** | **2.729 - 4.606** | **<0.0001** |
| HU-treated (yes vs WW; *n* = 659) * | **3.089** | **2.227 - 4.286** | **<0.0001** |
| RUX-treated (yes vs WW; *n* = 498) † | 1.479 | 0.925 - 2.365 | 0.1020 |
| Other MPN treatment (yes vs WW; *n* = 742) ‡ | **5.133** | **3.751 - 7.023** | **<0.0001** |
| Antithrombotic therapy (yes vs no; *n* = 1124) | **1.524** | **1.178 - 1.972** | **0.0013** |
| ASA (yes vs no; *n* = 921) | **1.630** | **1.244 - 2.137** | **0.0004** |
| P2Y12-antagonists (yes vs no; *n* = 366) § | 1.116 | 0.520 - 2.393 | 0.7785 |
| Anticoagulant-treated (yes vs no; *n* = 497) \|\| | 1.210 | 0.787 - 1.862 | 0.3854 |
| Multiple regression (n = 1087) ¶ | | | |
| ET vs MF | **1.598** | **1.140 - 2.239** | **0.0065** |
| MF vs PV | **0.474** | **0.335 - 0.672** | **<0.0001** |
| HU-treated (yes vs WW) | **2.750** | **1.971 - 3.838** | **<0.0001** |
| RUX-treated (yes vs WW) | **2.043** | **1.231 - 3.392** | **0.0057** |
| Other MPN treatment (yes vs WW) | **5.381** | **3.913 - 7.399** | **<0.0001** |
| By Diagnosis | | | |
| ET (*n* = 308) | | | |
| Arterial hypertension (yes vs no) | **1.738** | **1.029 - 2.936** | **0.0387** |
| HU-treated (yes vs WW) | **2.185** | **1.131 -4.218** | **0.0199** |
| Other MPN treatment (yes vs WW) | **7.337** | **4.043 - 13.315** | **<0.0001** |
| PV (*n* = 227) | | | |
| Diabetes mellitus (yes vs no) | **0.231** | **0.081 - 0.662** | **0.0064** |
| Arterial hypertension (yes vs no) | **2.082** | **1.089 - 3.981** | **0.0266** |
| HU-treated (yes vs WW) | **4.251** | **1.995 - 9.058** | **0.0002** |
| RUX-treated (yes vs WW) | **5.036** | **1.038 - 24.427** | **0.0448** |
| Other MPN treatment (yes vs WW) | **16.813** | **6.634 - 42.555** | **<0.0001** |
| MF (*n* = 324) | | | |
| HU-treated (yes vs WW) | **3.249** | **1.446 - 7.297** | **0.0043** |
| Other MPN treatment (yes vs WW) | **3.440** | **1.938 - 6.106** | **<0.0001** |

* Hydroxyurea. † Ruxolitinib. ‡ Anagrelide, IMIDs, interferon. § P2Y12-antagonist-treated: clopidogrel/ticagrelor/prasugrel. | | Anitcoagulant-treated: vitamin K-antagonist/dabigatran/rivaroxaban/apixaban/heparin/fondaparinux. ¶ Initial model including diagnosis, diabetes mellitus, leukocytes, arterial hypertension, MPN-therapy, and antithrombotic therapy. Bolded values indicate significant odds ratios and significant *P* values.

**Table S11.** Frequencies of thromboses across eGFR groups by risk factor.

| Risk factor (*n* = 1420) (*n*, % row, % column) | TE | eGFR1 | eGFR2 | eGFR3 | Total | *p* |
|---|---|---|---|---|---|---|
| ET | | | | | | 0.3726 |
| | | 33 | 75 | 34 | 142 | |
| | yes | 23.24 | 52.82 | 23.94 | 26.01 | |
| | | 25.58 | 24.35 | 31.19 | | |
| | | 96 | 233 | 75 | 404 | |
| | no | 23.76 | 57.67 | 18.56 | 73.99 | |
| | | 74.42 | 75.65 | 68.81 | | |
| PV | | | | | | **0.0243** |

| **Risk factor (*n* = 1420)**<br>**(*n*, % row, % column)** | **TE** | **eGFR1** | **eGFR2** | **eGFR3** | **Total** | ***p*** |
|---|---|---|---|---|---|---|
| | yes | 31<br>19.25<br>30.39 | 86<br>53.42<br>30.82 | 44<br>27.33<br>45.36 | 161<br>33.68 | |
| | no | 71<br>2.40<br>69.61 | 193<br>60.88<br>69.18 | 53<br>16.72<br>54.64 | 317<br>66.32 | |
| MF | | | | | | 0.3616 |
| | yes | 16<br>14.41<br>21.92 | 60<br>54.05<br>28.30 | 35<br>31.53<br>31.53 | 111<br>28.03 | |
| | no | 57<br>20.00<br>78.08 | 152<br>53.33<br>71.70 | 76<br>26.67<br>68.47 | 285<br>71.97 | |
| PMF | | | | | | 0.3351 |
| | yes | 15<br>16.67<br>23.08 | 47<br>52.22<br>28.48 | 28<br>31.11<br>34.15 | 90<br>28.85 | |
| | no | 50<br>22.52<br>76.92 | 118<br>53.15<br>71.52 | 54<br>24.32<br>65.85 | 222<br>71.15 | |
| Post-ET-MF | | | | | | >0.9999 |
| | yes | 1<br>7.69<br>16.67 | 7<br>53.85<br>28.00 | 5<br>38.46<br>25.00 | 13<br>25.49 | |
| | no | 5<br>13.16<br>83.33 | 18<br>47.37<br>72.00 | 15<br>39.47<br>75.00 | 38<br>74.51 | |
| Post-PV-MF | | | | | | >0.9999 |
| | yes | 0<br>0.00<br>0.00 | 6<br>75.00<br>27.27 | 2<br>25.00<br>22.22 | 8<br>24.24 | |
| | no | 2<br>8.00<br>100.00 | 16<br>64.00<br>72.73 | 7<br>28.00<br>77.78 | 25<br>75.76 | |
| Female gender | | | | | | **0.0321** |
| | yes | 34<br>17.26<br>23.61 | 101<br>51.27<br>25.06 | 62<br>31.47<br>34.64 | 197<br>27.13 | |
| | no | 110<br>20.79<br>76.39 | 302<br>57.09<br>74.94 | 117<br>22.12<br>65.36 | 529<br>72.87 | |
| Male gender | | | | | | 0.2565 |
| | yes | 46<br>21.20<br>28.75 | 120<br>55.30<br>30.30 | 51<br>23.50<br>36.96 | 217<br>31.27 | |
| | no | 114<br>23.90<br>71.25 | 276<br>57.86<br>69.70 | 87<br>18.24<br>63.04 | 477<br>68.73 | |
| Age at diagnosis >60, years | | | | | | 0.3837 |
| | yes | 23<br>12.04<br>28.75 | 95<br>49.74<br>29.87 | 73<br>38.22<br>35.10 | 191<br>31.52 | |
| | no | 57<br>13.73<br>71.25 | 223<br>53.73<br>70.13 | 135<br>32.53<br>64.90 | 415<br>68.48 | |
| Age at diagnosis ≤60, years | | | | | | **0.0364** |
| | yes | 56<br>25.23<br>25.11 | 126<br>56.76<br>26.20 | 40<br>18.02<br>37.74 | 222<br>27.41 | |
| | no | 167<br>28.40<br>74.89 | 355<br>60.37<br>73.80 | 66<br>11.22<br>62.26 | 588<br>72.59 | |
| Age at creatinine test >60, years | | | | | | 0.0903 |
| | yes | 32<br>13.33<br>31.07 | 115<br>47.92<br>27.64 | 93<br>38.75<br>35.63 | 240<br>30.77 | |
| | no | 71<br>13.15<br>68.93 | 301<br>55.74<br>72.36 | 168<br>31.11<br>64.37 | 540<br>69.23 | |
| Age at creatinine test ≤60, years | | | | | | 0.2006 |

| **Risk factor (*n* = 1420)**<br>**(*n*, % row, % column)** | **TE** | **eGFR1** | **eGFR2** | **eGFR3** | **Total** | ***p*** |
|---|---|---|---|---|---|---|
| | yes | 48<br>27.59<br>23.88 | 106<br>60.92<br>27.68 | 20<br>35.71 | 174<br>27.19 | |
| | no | 153<br>32.83<br>76.12 | 277<br>59.44<br>72.32 | 36<br>7.73<br>64.29 | 466<br>72.81 | |
| JAK2V617F mutation = yes | | | | | | **0.0437** |
| | yes | 62<br>20.00<br>29.81 | 161<br>51.94<br>30.21 | 87<br>28.06<br>39.01 | 310<br>32.16 | |
| | no | 146<br>22.32<br>70.19 | 372<br>56.88<br>69.79 | 136<br>20.80<br>60.99 | 654<br>67.84 | |
| JAK2V617F mutation = no | | | | | | **0.0322** |
| | yes | 13<br>16.46<br>15.66 | 45<br>56.96<br>21.84 | 21<br>26.58<br>33.87 | 79<br>22.51 | |
| | no | 70<br>25.74<br>84.34 | 161<br>59.19<br>78.16 | 41<br>15.07<br>66.13 | 272<br>77.49 | |
| CALR mutation = yes | | | | | | 0.1596 |
| | yes | 3<br>15.79<br>7.69 | 15<br>78.95<br>18.29 | 1<br>5.26<br>5.56 | 19<br>13.67 | |
| | no | 36<br>30.00<br>92.31 | 67<br>55.83<br>81.71 | 17<br>14.17<br>94.44 | 120<br>86.33 | |
| CALR mutation = no | | | | | | 0.6165 |
| | yes | 25<br>20.49<br>30.49 | 64<br>52.46<br>32.16 | 33<br>27.05<br>37.08 | 122<br>32.97 | |
| | no | 57<br>22.98<br>69.51 | 135<br>54.44<br>67.84 | 56<br>22.58<br>62.92 | 248<br>67.03 | |
| Diabetes = yes | | | | | | 0.6957 |
| | yes | 8<br>24.24<br>38.10 | 14<br>42.42<br>36.84 | 11<br>33.33<br>28.95 | 33<br>34.02 | |
| | no | 13<br>20.31<br>61.90 | 24<br>37.50<br>63.16 | 27<br>42.19<br>71.05 | 64<br>65.98 | |
| Diabetes = no | yes | 65<br>19.23<br>26.42 | 189<br>55.92<br>28.17 | 84<br>24.85<br>35.44 | 338<br>29.29 | 0.0573 |
| | no | 181<br>22.18<br>73.58 | 482<br>59.07<br>71.83 | 153<br>18.75<br>64.56 | 816<br>70.71 | |
| Arterial hypertension = yes | | | | | | 0.3167 |
| | yes | 28<br>15.14<br>34.57 | 90<br>48.65<br>38.30 | 67<br>36.22<br>44.08 | 185<br>39.53 | |
| | no | 53<br>18.73<br>65.43 | 145<br>51.24<br>61.7 | 85<br>30.04<br>55.92 | 283<br>60.47 | |
| Arterial hypertension = no | yes | 35<br>27.34<br>25.36 | 75<br>58.59<br>26.32 | 18<br>14.06<br>27.69 | 128<br>26.23 | 0.9387 |
| | no | 103<br>28.61<br>74.64 | 210<br>58.33<br>73.68 | 47<br>13.06<br>72.31 | 360<br>73.77 | |
| Hyperlipidemia = yes | | | | | | 0.0714 |
| | yes | 0<br>0.00<br>0.00 | 7<br>53.85<br>70.0 | 6<br>46.15<br>75.00 | 13<br>61.90 | |
| | no | 3<br>37.50<br>100.0 | 3<br>37.50<br>30.006 | 2<br>25.00<br>25.00 | 8<br>38.10 | |

| **Risk factor ($n$ = 1420) ($n$, % row, % column)** | **TE** | **eGFR1** | **eGFR2** | **eGFR3** | **Total** | ***p*** |
|---|---|---|---|---|---|---|
| Hyperlipidemia = no | yes | 13<br>22.81<br>26.53 | 35<br>61.40<br>38.89 | 9<br>15.79<br>42.86 | 57<br>35.63 | 0.2639 |
| | no | 36<br>34.95<br>73.47 | 55<br>53.40<br>61.11 | 12<br>11.65<br>57.14 | 103<br>64.38 | |
| Leukocytes >8.4, G/L | | | | | | **0.0011** |
| | yes | 28<br>14.43<br>19.72 | 97<br>50.00<br>26.01 | 69<br>35.57<br>37.30 | 194<br>27.71 | |
| | no | 114<br>22.53<br>80.28 | 276<br>54.55<br>73.99 | 116<br>22.92<br>62.70 | 506<br>72.29 | |
| Leukocytes ≤8.4, G/L | | | | | | 0.6514 |
| | yes | 52<br>23.64<br>32.10 | 124<br>56.36<br>29.81 | 44<br>20.00<br>33.85 | 220<br>31.07 | |
| | no | 110<br>22.54<br>67.90 | 292<br>59.84<br>70.19 | 86<br>17.62<br>66.15 | 488<br>68.93 | |
| Platelets >483, G/L | | | | | | **0.0336** |
| | yes | 31<br>18.02<br>20.26 | 96<br>55.81<br>23.59 | 45<br>26.16<br>32.85 | 172<br>24.68 | |
| | no | 122<br>23.24<br>79.74 | 311<br>59.24<br>76.41 | 92<br>17.52<br>67.15 | 525<br>75.32 | |
| Platelets ≤483, G/L | | | | | | 0.4083 |
| | yes | 49<br>20.59<br>32.67 | 121<br>50.84<br>32.70 | 68<br>28.57<br>38.20 | 238<br>34.10 | |
| | no | 101<br>21.96<br>67.33 | 249<br>54.13<br>67.30 | 110<br>23.91<br>61.80 | 460<br>65.90 | |
| Uric acid >5.7, mg/dL | | | | | | 0.3087 |
| | yes | 22<br>14.86<br>28.95 | 72<br>48.65<br>28.02 | 54<br>36.49<br>35.06 | 148<br>30.39 | |
| | no | 54<br>15.93<br>71.05 | 185<br>54.57<br>71.98 | 100<br>29.50<br>64.94 | 339<br>69.61 | |
| Uric acid ≤5.7, mg/dL | | | | | | 0.2209 |
| | yes | 35<br>24.48<br>23.97 | 84<br>58.74<br>27.01 | 24<br>16.78<br>35.29 | 143<br>27.24 | |
| | no | 111<br>29.06<br>76.03 | 227<br>59.42<br>72.99 | 44<br>11.52<br>64.71 | 382<br>72.76 | |
| Neutrophils >5.44, G/L | | | | | | **<0.0001** |
| | yes | 20<br>11.83<br>16.00 | 87<br>51.48<br>25.66 | 62<br>36.69<br>38.51 | 169<br>27.04 | |
| | no | 105<br>23.03<br>84.00 | 252<br>55.26<br>74.34 | 99<br>21.71<br>61.49 | 456<br>72.96 | |
| Neutrophils ≤5.44, G/L | | | | | | 0.7214 |
| | yes | 43<br>21.72<br>29.25 | 117<br>59.09<br>31.62 | 38<br>19.19<br>33.93 | 198<br>31.48 | |
| | no | 104<br>24.13<br>70.75 | 253<br>58.70<br>68.38 | 74<br>17.17<br>66.07 | 431<br>68.52 | |
| Monocytes >0.55, G/L | | | | | | **0.0073** |
| | yes | 27<br>14.06<br>20.00 | 102<br>53.13<br>29.82 | 63<br>32.81<br>36.42 | 192<br>29.54 | |
| | no | 108<br>23.58<br>80.00 | 240<br>52.40<br>70.18 | 110<br>24.02<br>63.58 | 458<br>70.46 | |
| Monocytes ≤0.55, G/L | | | | | | |

| **Risk factor (*n* = 1420)<br>(*n*, % row, % column)** | **TE** | **eGFR1** | **eGFR2** | **eGFR3** | **Total** | ***p*** |
|---|---|---|---|---|---|---|
| | yes | 41<br>21.58<br>27.33 | 108<br>56.84<br>27.62 | 41<br>21.58<br>33.88 | 190<br>28.70 | 0.3775 |
| | no | 109<br>23.09<br>72.67 | 283<br>59.96<br>72.38 | 80<br>16.95<br>66.12 | 472<br>71.30 | |
| LDH >267.5, U/L | | | | | | 0.0749 |
| | yes | 34<br>15.74<br>26.56 | 113<br>52.31<br>30.62 | 69<br>31.94<br>38.12 | 216<br>31.86 | |
| | no | 94<br>20.35<br>73.44 | 256<br>55.41<br>69.38 | 112<br>24.24<br>61.88 | 462<br>68.14 | |
| LDH ≤267.5, U/L | | | | | | 0.1541 |
| | yes | 42<br>22.70<br>25.61 | 102<br>55.14<br>25.82 | 41<br>22.16<br>34.45 | 185<br>27.29 | |
| | no | 122<br>24.75<br>74.39 | 293<br>59.43<br>74.18 | 78<br>15.82<br>65.55 | 493<br>72.71 | |
| CRP >1.5, mg/L | | | | | | 0.1473 |
| | yes | 10<br>16.39<br>28.57 | 27<br>44.26<br>31.76 | 24<br>39.34<br>46.15 | 61<br>35.47 | |
| | no | 25<br>22.52<br>71.43 | 58<br>52.25<br>68.24 | 28<br>25.23<br>53.85 | 111<br>64.53 | |
| CRP ≤1.5, mg/L | | | | | | 0.1707 |
| | yes | 9<br>15.52<br>20.45 | 37<br>63.79<br>33.94 | 12<br>20.69<br>38.71 | 58<br>31.52 | |
| | no | 35<br>27.78<br>79.55 | 72<br>57.14<br>66.06 | 19<br>15.08<br>61.29 | 126<br>68.48 | |

Bolded values indicate significant *P* values.

**Table S12.** Distribution of arterial and venous thromboembolism across eGFR groups.

| **Type of thromboembolism (*n* = 1409)<br>(*n*, % row, % column)** | **eGFR1** | **eGFR2** | **eGFR3** | **Total** | ***p*** |
|---|---|---|---|---|---|
| Arterial | 43<br>18.86<br>14.14 | 122<br>53.51<br>15.44 | 63<br>27.63<br>20.00 | 228<br>100.00<br>16.18 | |
| Venous including splanchnic | 34<br>24.29<br>11.18 | 72<br>51.43<br>9.11 | 34<br>24.29<br>10.79 | 140<br>100.00<br>9.94 | |
| Arterial and venous including splanchnic | 3<br>8.57<br>0.99 | 18<br>51.43<br>2.28 | 14<br>40.00<br>4.44 | 35<br>100.00<br>2.48 | |
| No thromboembolism | 224<br>22.27<br>73.68 | 578<br>57.46<br>73.16 | 204<br>20.28<br>64.76 | 1006<br>100.00<br>71.40 | |
| *Comparisons between type of thromboembolism* | | | | | |
| Arterial vs venous/splanchnic vs no vs arterial + venous | | | | | 0.0174 |
| Arterial vs venous/splanchnic vs arterial + venous | | | | | 0.5262 |
| Arterial vs venous/splanchnic vs no (excl. arterial + venous) | | | | | 0.3324 |
| Arterial vs venous (excl. arterial + venous) | | | | | >0.9999 |

Bolded values indicate significant *P* values.

**Table S13.** Frequency of HU, RUX, anagrelide/IMIDs/interferon, and no therapy across eGFR groups.

| **Sample (*n* = 1215)<br>(*n*, % row, % column)** | **Therapy<br>(*n*, %)** | **eGFR1** | **eGFR2** | **eGFR3** | ***p*** | |
|---|---|---|---|---|---|---|
| | | | | | **overall** | **pairwise *** |
| All patients (*n* = 1215) | | | | | **<0.0001** | |
| | HU only<br>(256, 21.07) | 41<br>16.02<br>15.71 | 155<br>60.55<br>22.79 | 60<br>23.44<br>21.90 | | **0.0021** |
| | RUX only | 14 | 49 | 31 | | **0.0002** |

| Sample ($n$ = 1215) (*n*, % row, % column) | Therapy (*n*, %) | eGFR1 | eGFR2 | eGFR3 | *p* overall | *p* pairwise * |
|---|---|---|---|---|---|---|
| | (94, 7.74) | 14.89 | 52.13 | 32.98 | | |
| | | 5.36 | 7.21 | 11.31 | | |
| | other | 72 | 167 | 102 | | |
| | (341, 28.07) | 21.11 | 48.97 | 29.91 | | |
| | | 27.59 | 24.56 | 37.23 | | |
| | Multiple therapies | 49 | 119 | 82 | | |
| | (250, 20.58) | 19.60 | 47.60 | 32.80 | | **<0.0001** |
| | | 18.77 | 17.50 | 29.93 | | |
| | WW | 134 | 309 | 81 | | |
| | (524, 43.13) | 25.57 | 58.97 | 15.46 | | |
| | | 51.34 | 45.44 | 29.56 | | |
| ET ($n$ = 471) | | | | | **0.0001** | |
| | HU only | 17 | 52 | 19 | | |
| | (88, 18.68) | 19.32 | 59.09 | 21.59 | | **0.0227** |
| | | 14.66 | 19.48 | 21.59 | | |
| | RUX only | 0 | 3 | 2 | | |
| | (5, 1.06) | 0.00 | 60.00 | 40.00 | | 0.0942 |
| | | 0.00 | 1.12 | 2.27 | | |
| | other | 35 | 69 | 44 | | |
| | (148, 31.42) | 23.65 | 46.62 | 29.73 | | |
| | | 30.17 | 25.84 | 50.00 | | |
| | Multiple therapies | 20 | 38 | 30 | | |
| | (88, 18.68) | 22.73 | 43.18 | 34.09 | | **0.0006** |
| | | 17.24 | 14.23 | 34.09 | | |
| | WW | 64 | 143 | 23 | | |
| | (230, 48.83) | 27.83 | 62.17 | 10.00 | | |
| | | 55.17 | 53.56 | 26.14 | | |
| PV ($n$ = 395) | | | | | 0.5082 | |
| | HU only | 22 | 81 | 32 | | |
| | (135, 34.18) | 16.30 | 60.00 | 23.70 | | 0.6992 |
| | | 28.57 | 35.06 | 36.78 | | |
| | RUX only | 2 | 6 | 3 | | |
| | (11, 2.78) | 18.18 | 54.55 | 27.27 | | >0.9999 |
| | | 2.60 | 2.60 | 3.45 | | |
| | other | 22 | 45 | 23 | | |
| | (90, 22.78) | 24.44 | 50.00 | 25.56 | | |
| | | 28.57 | 19.48 | 26.44 | | |
| | Multiple therapies | 16 | 39 | 22 | | |
| | (77, 19.49) | 20.78 | 50.65 | 28.57 | | 0.6879 |
| | | 20.78 | 16.88 | 25.29 | | |
| | WW | 31 | 99 | 29 | | |
| | (159, 40.25) | 19.50 | 62.26 | 18.24 | | |
| | | 40.26 | 42.86 | 33.33 | | |
| MF ($n$ = 349) | | | | | **0.0103** | |
| | HU only | 2 | 22 | 9 | | |
| | (33, 9.46) | 6.06 | 66.67 | 27.27 | | **0.0351** |
| | | 2.94 | 12.09 | 9.09 | | |
| | RUX only | 12 | 40 | 26 | | |
| | (78, 22.35) | 15.38 | 51.28 | 33.33 | | 0.0587 |
| | | 17.65 | 21.98 | 26.26 | | |
| | other | 15 | 53 | 35 | | |
| | (103, 29.51) | 14.56 | 51.46 | 33.98 | | |
| | | 22.06 | 29.12 | 35.35 | | |
| | Multiple therapies | 13 | 42 | 30 | | |
| | (85, 24.36) | 15.29 | 49.41 | 35.29 | | 0.6447 |
| | | 19.12 | 23.08 | 30.30 | | |
| | WW | 39 | 67 | 29 | | |
| | (135, 38.68) | 28.89 | 49.63 | 21.48 | | |
| | | 57.35 | 36.81 | 29.29 | | |

* Therapy compared to WW. Bolded values indicate significant *P* values.

**Table S14.** Frequency of ASA, P2Y12-antagonists, or anticoagulation therapy across eGFR groups.

| Sample ($n$ = 1251) (*n*, % row, % column) | Therapy (*n*, %) | eGFR1 | eGFR2 | eGFR3 | *p* overall | *p* pairwise * |
|---|---|---|---|---|---|---|
| All patients ($n$ = 1251) | | | | | **0.0033** | |
| | ASA | 128 | 395 | 131 | | |
| | (654, 52.28) | 19.57 | 60.40 | 20.03 | | 0.3387 |
| | | 48.48 | 56.19 | 46.13 | | |
| | P2Y12-antagonists | 5 | 17 | 8 | | >0.9999 |

| **Sample ($n$ = 1251) ($n$, % row, % column)** | **Therapy ($n$, %)** | **eGFR1** | **eGFR2** | **eGFR3** | ***p*** **overall** | ***p*** **pairwise *** |
|---|---|---|---|---|---|---|
| | (30, 2.40) | 16.67 | 56.67 | 26.67 | | |
| | | 1.89 | 2.42 | 2.82 | | |
| | Anticoagulant-treated (122, 9.75) | 28 | 51 | 43 | | |
| | | 22.95 | 41.80 | 35.25 | | **0.0237** |
| | | 10.61 | 7.25 | 15.14 | | |
| | no (375, 29.98) | 91 | 203 | 81 | | |
| | | 24.27 | 54.13 | 21.60 | | |
| | | 34.47 | 28.88 | 28.52 | | |
| ET ($n$ = 483) | | | | | 0.0930 | |
| | ASA (272, 56.31) | 63 | 156 | 53 | | |
| | | 23.16 | 57.35 | 19.49 | | 0.1953 |
| | | 52.07 | 58.43 | 55.79 | | |
| | P2Y12-antagonists (19, 3.93) | 4 | 9 | 6 | | |
| | | 21.05 | 47.37 | 31.58 | | 0.2967 |
| | | 3.31 | 3.37 | 6.32 | | |
| | Anticoagulant-treated (35, 7.04) | 8 | 16 | 10 | | |
| | | 23.53 | 47.06 | 29.41 | | 0.1968 |
| | | 6.61 | 5.99 | 10.53 | | |
| | no (124, 25.67) | 41 | 67 | 16 | | |
| | | 33.06 | 54.03 | 12.90 | | |
| | | 33.88 | 25.09 | 16.84 | | |
| PV ($n$ = 415) | | | | | 0.1193 | |
| | ASA (254, 61.20) | 51 | 157 | 46 | | |
| | | 20.08 | 61.81 | 18.11 | | 0.4626 |
| | | 62.96 | 64.08 | 51.69 | | |
| | P2Y12-antagonists (7, 1.69) | 1 | 6 | 0 | | |
| | | 14.29 | 85.71 | 0.00 | | 0.7311 |
| | | 1.23 | 2.45 | 0.00 | | |
| | Anticoagulant-treated (59, 14.22) | 14 | 26 | 19 | | |
| | | 23.73 | 44.07 | 32.20 | | 0.5388 |
| | | 17.28 | 10.61 | 21.35 | | |
| | no (73, 17.59) | 10 | 43 | 20 | | |
| | | 13.70 | 58.90 | 27.40 | | |
| | | 12.35 | 17.55 | 22.47 | | |
| MF ($n$ = 353) | | | | | **0.0047** | |
| | ASA (128, 36.26) | 14 | 82 | 32 | | |
| | | 10.94 | 64.06 | 25.00 | | 0.0729 |
| | | 22.58 | 42.93 | 32.00 | | |
| | P2Y12-antagonists (4, 1.13) | 0 | 2 | 2 | | |
| | | 0.00 | 50.00 | 50.00 | | >0.9999 |
| | | 0.00 | 1.05 | 2.00 | | |
| | Anticoagulant-treated (29, 8.22) | 6 | 9 | 14 | | |
| | | 20.69 | 31.03 | 48.28 | | 0.0933 |
| | | 9.68 | 4.71 | 14.00 | | |
| | no (178, 50.42) | 40 | 93 | 45 | | |
| | | 22.47 | 52.25 | 25.28 | | |
| | | 64.52 | 48.69 | 45.00 | | |

* Compared to no therapy. Bolded values indicate significant *P* values.

**Publisher's Note:** MDPI stays neutral with regard to jurisdictional claims in published maps and institutional affiliations.

© 2021 by the authors. Licensee MDPI, Basel, Switzerland. This article is an open access article distributed under the terms and conditions of the Creative Commons Attribution (CC BY) license (http://creativecommons.org/licenses/by/4.0/).
